# Validating ORR and PFS as surrogate endpoints in phase II and III clinical trials for NSCLC patients: difference exists in the strength of surrogacy in various trial settings

**DOI:** 10.1186/s12885-022-10046-z

**Published:** 2022-09-29

**Authors:** Tiantian Hua, Yuan Gao, Ruyang Zhang, Yongyue Wei, Feng Chen

**Affiliations:** 1 Present Address: Department of Epidemiology and Health Statistics, School of Public Health, Southeast University, Nanjing, China; 2 Department of Biostatistics, School of Public Health, Nanjing Medical University, Nanjing, China

**Keywords:** Surrogate endpoint, Meta-analysis, Non-small cell lung cancer, Targeted therapy, Immunotherapy

## Abstract

**Objective:**

This study aims to systematically validate the performance of surrogate endpoints in phase II and III clinical trials for NSCLC patients under various trial settings.

**Methods:**

A literature search retrieved all registered phase II and III trials of NSCLC patients in which OS, with at least one of ORR and PFS, were reported. Associations between surrogate and true endpoints were assessed on two levels. On the arm level, three pairs of correlations, i.e., ORR vs. median OS, ORR vs. median PFS, and median PFS vs. median OS, were analysed using Spearman’s rho. On the trial level, similarly, three pairs of correlations, i.e., ΔORR vs. HR of OS, ΔORR vs. HR of PFS, and HR of PFS vs. HR of OS, were analysed using Spearman’s rho and weighted linear regression model respectively. Finally, sensitivity analyses were performed to explore surrogacy under various trial settings.

**Results:**

At arm level, three pairs of correlations are all high (Spearman’s rho = 0.700, 0.831, 0.755, respectively). At trial level, there is a low correlation between ΔORR and HR of OS, a high correlation between ΔORR and HR of PFS and a moderate correlation between HR of PFS and HR of OS (Spearman’s rho = 0.462, 0.764, 0.584, respectively). In the sensitivity analysis, we find correlations between surrogate and true endpoints vary by different trial settings. It is noteworthy that the strength of surrogacy of these intermediate endpoints in targeted therapy is greater than that in immunotherapy.

**Conclusion:**

According to the arm-level and trial level-analysis, we suggest that in phase II and III trials of targeted therapy and immunotherapy for NSCLC patients: 1) ORR lacks validity for the surrogacy of OS, excluding in first-line therapy, and 2) ORR may be an appropriate surrogate endpoint for PFS, and 3) PFS may be considered a modest surrogacy for OS, with better performance in first-line therapy trials. Moreover, to provide more convincing evidence of surrogacy of the surrogate endpoints, patient-level analyses are in desperate need.

**Supplementary Information:**

The online version contains supplementary material available at 10.1186/s12885-022-10046-z.

## Background

Most recently, targeted therapy and immunotherapy have sprung up in cancer therapy [1]. The development of targeted therapy has advanced the therapeutic strategy from conventional chemo-based and radiation-based therapy to genetic alteration-guided targeted therapy [2, 3]. Meanwhile, the advent of immunotherapy leads to greater availability of effective subsequent treatments and extended survival in previously treated advanced non-small cell lung cancer (NSCLC), of which a good example is the success of clinical trials for PD-1/PD-L1 inhibitor in tumour treatment [4, 5].

In oncology trials, intermediate/surrogate endpoints are often used as primary endpoints instead of true endpoints such as the overall survival (OS) over the past decade [6]. Surrogate endpoints are not intrinsically beneficial to patients but are designed to be easier, faster and cheaper to measure than clinically meaningful outcomes because they can reduce the sample size, shorten the duration and save the cost of trials [7, 8]. Although there have been many studies trying to determine the surrogacy of intermediate endpoints, the results didn’t reach a consensus [9–15]. Besides, evaluating the surrogacy of the intermediate endpoints in these new domains, such as immunotherapy and targeted therapy, raises a to-be-solved challenge.

We, therefore, performed a broad-based structured review and meta-analysis of registered phase II and III NSCLC trials with immunotherapy and/or targeted therapy. The objective of this research was to find the appropriacy of surrogate endpoints in NSCLC studies under various trial settings (e.g. line of therapy, trial phase, blinding and therapy type), to give suggestions on which surrogate endpoints should be used under certain trial conditions.

## Methods

### Search strategy

**Fig. 1:**
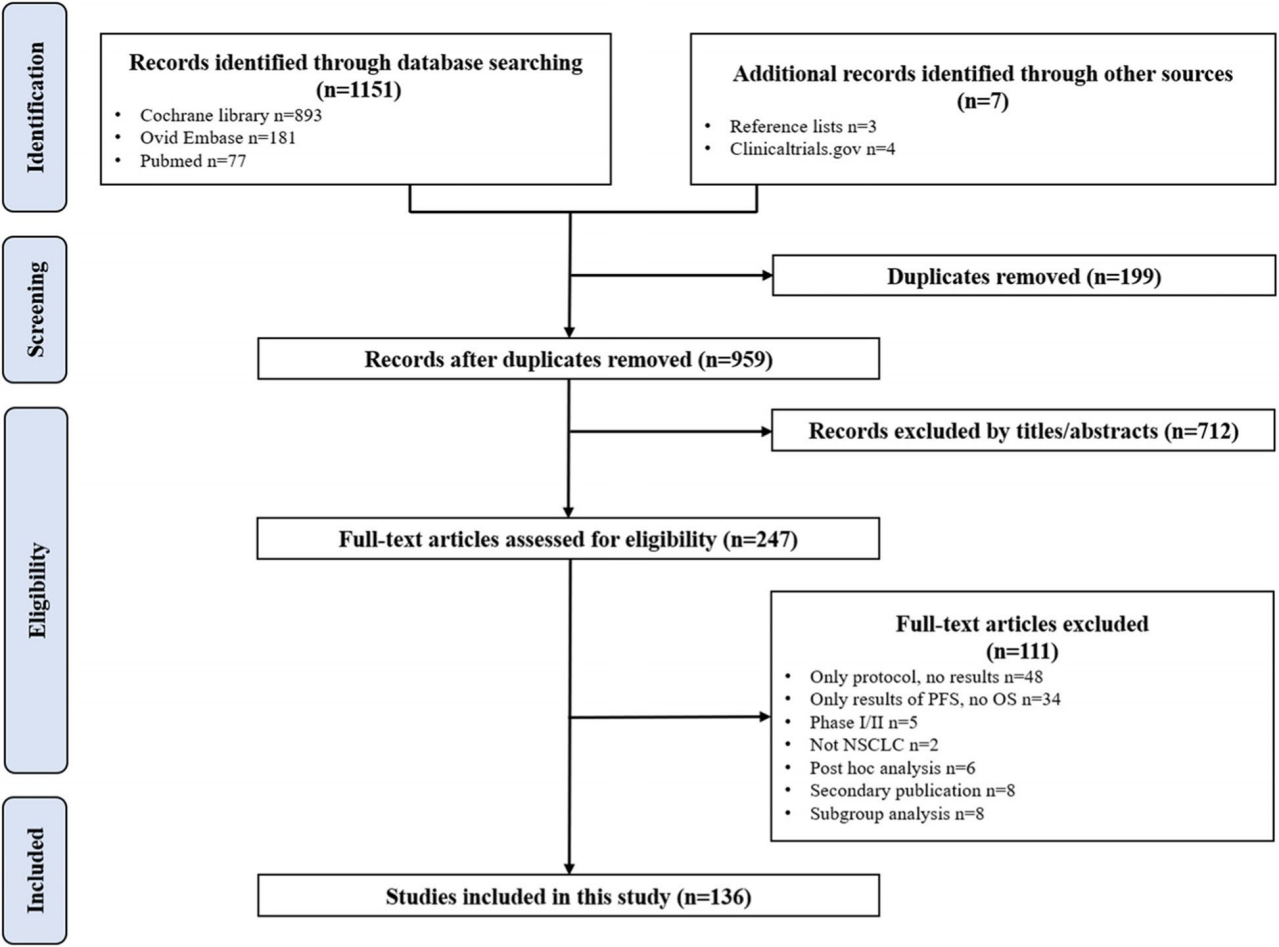
PRISMA flow diagram for study inclusion

We conducted this analysis in compliance with the Preferred Reporting Items for Systematic Reviews and Meta-Analyses (PRISMA) Statement [16]. Three databases (PubMed, EMBASE, Cochrane Library) were searched with time ranged from January 2000 to May 2021. Search terms included cancer terms AND therapy terms AND terms for PFS (Progression Free Survival, PFS), ORR (Objective Response Rate, ORR) and/or OS (Overall Survival, OS) AND terms for endpoint and/or surrogate. Search results were limited to the English language and in-human studies. The detailed Cochrane Library search strategy is provided in Additional file 1. The PRISMA flow diagram is shown in Fig. 1.

### Inclusion and exclusion criteria

Inclusion was restricted to registered clinical trials reporting results of OS, with either PFS or ORR, or both. The included studies should report the NCT number. Protocols and ongoing trials without results were excluded.

### Study selection and data extraction

Titles and abstracts of articles retrieved by the search were examined by two independent reviewers, followed by a discussion to ensure consistency in the selection decisions. Full texts were examined by one reviewer and a subset was checked by a second reviewer, with any discrepancies resolved through discussion.

Data were extracted by one reviewer and checked by a second reviewer. For eligible studies, the following characteristics were extracted: authors, publication year, number of patients included, type of lung cancer, stage of lung cancer, therapy type, NCT number, surrogate and final endpoints analysed, line of therapy, phase of the trial, results including ORR, median PFS and median OS of each arm, and ΔORR, HR of PFS and HR of OS between arms. The summary of included articles is shown in Additional file 2.

### Data synthesis and analysis

According to the definition and validation criteria for surrogate endpoints [17, 18], two conditions are necessary to measure whether an intermediate endpoint can be an acceptable surrogacy for the true clinical endpoint. First, for the individual level, there must be a strong association between the surrogate endpoint and the true endpoint. Second, for the trial level, there must be a strong association between the treatment effect of the surrogate endpoint and that of the true endpoint [19]. However, the individual level data are usually not available, so we use arm level data as an alternative, which can also be seen in many other studies [20–23]. To describe the correlation strength between surrogate and true endpoints in this study, we divided values of the correlation coefficient into five levels, as shown in Table 1.

**Table 1:** Rule for interpreting the strength of a correlation coefficient

Size of Correlation (absolute value)	Interpretation
[0.90,1.00]	Very high positive (negative) correlation
[0.70,0.90)	High positive (negative) correlation
[0.50,0.70)	Moderate positive (negative) correlation
[0.30,0.50)	Low positive (negative) correlation
[0.00,0.30)	Negligible correlation

The Spearman rank correlation coefficient, r_s_, was used to measure the arm-level associations, which means correlation coefficients between ORR and median OS, between ORR and median PFS, and between median PFS and median OS were calculated from all arms of all included studies.

To assess trial-level associations, correlations between OR of ORR (Odds Ratio of ORR) and HR of OS (Hazard Ratio of OS), between OR of ORR and HR of PFS, and between HR of PFS (Hazard Ratio of OS) and HR of OS were needed. In light of the situation that few studies reported OR of ORR, we replaced the OR of ORR by subtracting the treatment’s ORR from the control’s ORR, which was defined as ΔORR. Similarly, the Spearman rank correlation coefficient r_s_ was used to evaluate the associations.

Correlation indicates the extent to which those two treatment effects move together, while regression allows to predict the long-term treatment effect (true endpoint) based on the short-term treatment effect (surrogate endpoint). Hence, to give an idea of how the later can help with predicting the former, we further implemented the linear regression models. Simple linear regression models, followed by multiple linear regression models (both weighted by sample size as in previous endpoint validation studies [24–27]), which were expected to control for specific trial setting factors including line of therapy, trial phase and masking, were fitted. Also, we used the surrogate threshold effect (STE) to evaluate the surrogacy of surrogate endpoints from a clinical point of view [28]. STE defines the minimum short-term (i.e. surrogate endpoint) treatment effect required to guarantee a non-zero long-term (i.e. true endpoint) treatment effect. For example, take a regression of HR of OS as y on HR of PFS as x, then the x-value of the intersection of the line y = 1 (which means zero long-term treatment effect) and the upper boundary of the 95% prediction interval stripe of the regression line is STE.

It should be noted that various trial settings, such as line of therapy, trial phase, masking and type of therapy might introduce bias into the quantitative relationship between surrogate and true endpoints, if any of these factors remained from the stepwise selection in the previous multiple regression. Thus, sensitivity analyses were performed to explore whether the associations showed homogeneity regardless of different trial settings.

All analyses were conducted using R version 4.1.0 (R Foundation for Statistical Computing, Vienna, Austria).

## Results

After the initial search, 1158 publications were identified for screening and 959 potentially relevant unduplicated publications were reviewed in detail for eligibility. Ultimately, a total of 136 eligible trials with a total of 350 arms (including arms of reported subgroup analysis, with details in Additional file 2) and 46,028 NSCLC patients were included in the study. The study selection process and reasons for exclusion at each stage are detailed in the PRISMA flow diagram Fig. 1.

Most studies focused on advanced NSCLC. Among all 136 trials, 69 (50.7%) of them were phase II studies, 2 (1.5%) of them were phase II/III studies, and 65 (47.8%) of them were phase III studies. Concerning masking, 38 trials were double-blinded, 1 trial was single-blinded, and 97 trials were open-labelled. Other study design characteristics needed for sensitivity analysis are summarised in Additional file 2.

In all 350 arms, 288 (82.3%) arms reported ORR, 331 (94.6%) arms reported median PFS, and 311 (88.9%) arms reported median OS. Meanwhile, in all 136 trials, 20 (14.7%) trials reported 25 ORs of ORR, 103 (75.7%) trials reported 147 HRs of PFS, and 101 (74.3%) trials reported 145 HRs of OS. The low percentage of trials reporting OR of ORR led to our decision that ΔORR was constructed to replace OR of ORR.

### Arm-level associations between surrogate and true endpoints

Of all 350 arms from 136 eligible trials, 254 arms had both ORR and median OS reported, 271 arms had both ORR and median PFS reported, and 306 arms had both median PFS and median OS reported. The median PFS averaged across all arms was 4.5 months (range from 1.0 to 24.5 months), and the median OS was 11.5 months (range from 2.8 to 51.3 months).

**Fig. 2:**
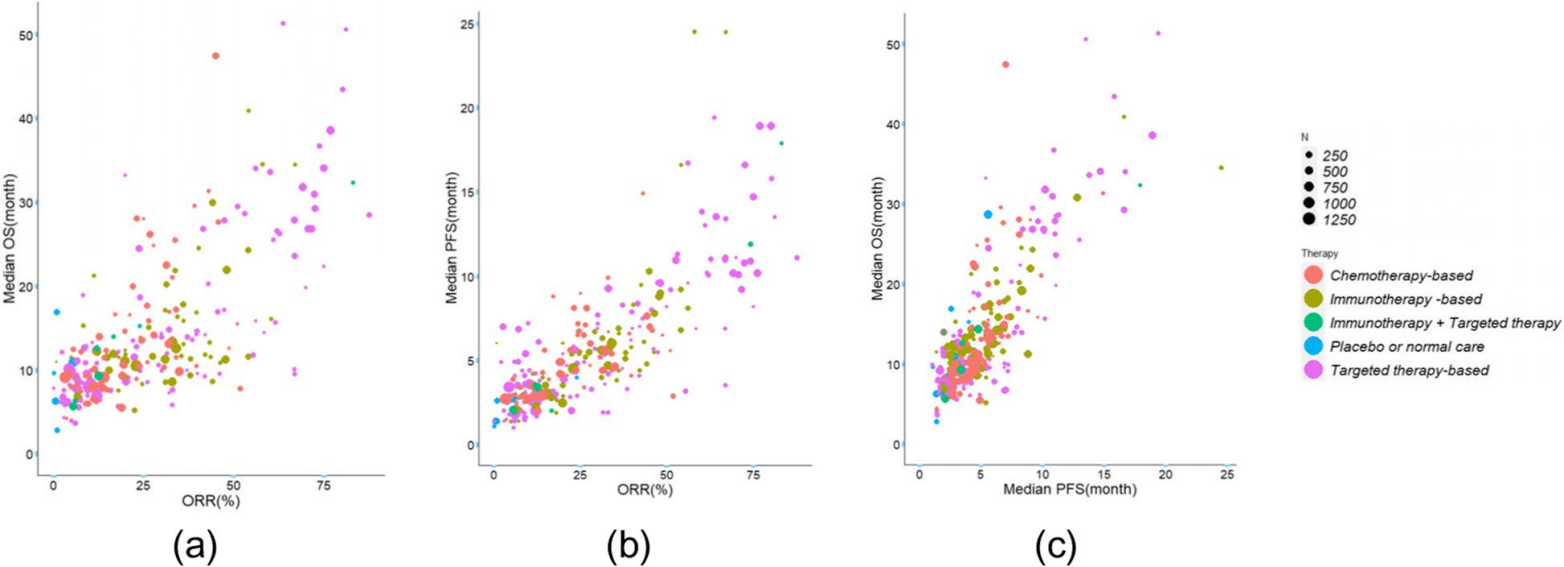
Arm-level scatter plot of surrogate and true endpoints. (a) Arm-level scatter plot of ORR and median OS. The Spearman’s rho coefficient is 0.700; (b) Arm-level scatter plot of ORR and median PFS. The Spearman’s rho coefficient is 0.831; (c) Arm-level scatter plot of median PFS and median OS. The Spearman’s rho coefficient is 0.755. Each arm is represented by a bubble whose radius is proportional to the study sample size. The bubble colour represents the therapy type of the arm

Figure 2 gives scatter plots of ORR vs. median OS, ORR vs. median PFS, and median PFS vs. median OS from all arms, with bubble radius representing the corresponding study sample size, and bubble colour representing the therapy type. The Spearman rank correlation coefficient suggested a high positive association between ORR and median OS (Spearman’s rho = 0.700, Table 2), a high positive association between ORR and median PFS (Spearman’s rho = 0.831, Table 2), and a high positive association between median PFS and median OS (Spearman’s rho = 0.755, Table 2).

**Table 2:** Arm-level association of all included arms

Surrogate endpoint	True endpoint	No. of arms	r_**s**_	95%CI
ORR	Median OS	254	0.700	0.631,0.758
ORR	Median PFS	271	0.831	0.790,0.864
Median PFS	Median OS	306	0.755	0.702,0.799

### Trial-level associations between surrogate and true endpoints

Of all 136 eligible trials, we extracted 112 pairs of ΔORR and HR of OS, 114 pairs of ΔORR and HR of PFS, and 138 pairs of HR of PFS and HR of OS (because some trials have more than one treatment arm). The ΔORR ranged from − 53.5 to 27.0% in all trials, with HR of PFS and HR of OS ranging from 0.16 to 2.00 and 0.22 to 1.78 respectively.

The Spearman correlation between the treatment effect of surrogate and true endpoints was first calculated, as shown in Table 3, which gave a low positive correlation of the pair ΔORR vs. HR of OS (Spearman’s rho = 0.462, Table 3), a high positive correlation of the pair ΔORR vs. HR of PFS (Spearman’s rho = 0.764, Table 3), and a moderate positive correlation of the pair HR of PFS vs. HR of OS (Spearman’s rho = 0.584, Table 3).

**Table 3:** Trial-level association of all included studies (Spearman’s rho)

Treatment effect of surrogate endpoint	Treatment effect of true endpoint	No. of trials	r_**s**_	95% CI
ΔORR	HR of OS	112	0.462	0.303, 0.597
ΔORR	HR of PFS	114	0.764	0.675, 0.831
HR of PFS	HR of OS	138	0.584	0.462, 0.684

Simple linear regression results are listed in Table 4. An increasement of 1 unit in ΔORR would lead to an increasement of 0.011 unit in log (HR of OS) (R^2^ = 0.261, Table 4) and 0.023 unit in log (HR of PFS) (R^2^ = 0.578, Table 4), while an increasement of 1 unit in log (HR of PFS) resulted in an increasement of 0.403 unit in log (HR of OS) (R^2^ = 0.360, Table 4).

**Table 4:** Trial-level associations of all included studies (Simple linear regression)

Treatment effect of surrogate endpoint	Treatment effect of true endpoint	No. of trials	Slope estimate	95% CI	R^**2**^	Adjusted R^**2**^	STE
ΔORR	HR of OS	112	0.011	0.007, 0.014	0.261	0.254	——^b^
ΔORR	HR of PFS^a^	114	0.023	0.019, 0.027	0.578	0.574	−26.30%
HR of PFS^a^	HR of OS^a^	138	0.403	0.313, 0.494	0.360	0.355	——^b^

Simple linear regression model form

log (HR of OS) = −0.062 + 0.011 * ΔORR;

log (HR of PFS) = − 0.103 + 0.023 * ΔORR;

log (HR of OS) = − 0.053 + 0.403 * log (HR of PFS)

^a^Natural log hazard ratio in the analysis

^b^The STE will be given only when the upper limit of r_s_ (in Table 3) is more than 0.7

The trial-level correlation between ΔORR and HR of PFS from the clinical point of view is reflected by a surrogate threshold effect (STE) equal to − 26.30%. In other words, if ORR of the control group minus ORR of the treatment group is smaller than − 26.30%, the HR of PFS will be both statistically and clinically significant, which suggests a reduction in the risk of progression in the experiment group.

Multiple linear regression results are listed in Table 5. Based on the least AIC criteria, trial setting factors were selected stepwise. Trial phase should be paid attention to when using ΔORR as a surrogate endpoint for HR of OS (log (HR of OS) = − 0.003 + 0.012 * ΔORR − 0.092 * phase) and using HR of PFS for HR of OS (log (HR of OS) = 0.009 + 0.418 * log (HR of PFS) - 0.072 * phase). Whether the trial is blinded also played a role in the surrogacy of ΔORR for HR of PFS (log (HR of PFS) = − 0.066 + 0.023 * ΔORR – 0.093 * masking).

**Table 5:** Trial-level association of all included studies (Multiple linear regression, adjusted for line, phase and masking^b^)

Treatment effect of surrogate endpoint	Treatment effect of true endpoint	No. of trials	Slope estimate	95% CI	R^**2**^	Adjusted R^**2**^
ΔORR	HR of OS^a^	103	0.012	0.008, 0.015	0.313	0.299
ΔORR	HR of PFS^a^	104	0.023	0.119, 0.027	0.571	0.562
HR of PFS^a^	HR of OS^a^	125	0.418	0.312, 0.524	0.393	0.380

Multivariable linear regression model form:

Log (HR of OS) = − 0.003 + 0.012 * ΔORR − 0.092 * phase;

Log (HR of PFS) = − 0.066 + 0.023 * ΔORR – 0.093 * masking;

Log (HR of OS) = 0.009 + 0.418 * log (HR of PFS) - 0.072 * phase

^a^Natural log hazard ratio in the analysis

^b^Code for line, phase and masking in the regression:

Line: First-line = 0, ≥2nd-line = 1

Phase: Phase II = 0, Phase III = 1

Masking: Open-label = 0, Double-blind = 1

### Additional analysis

**Fig. 3:**
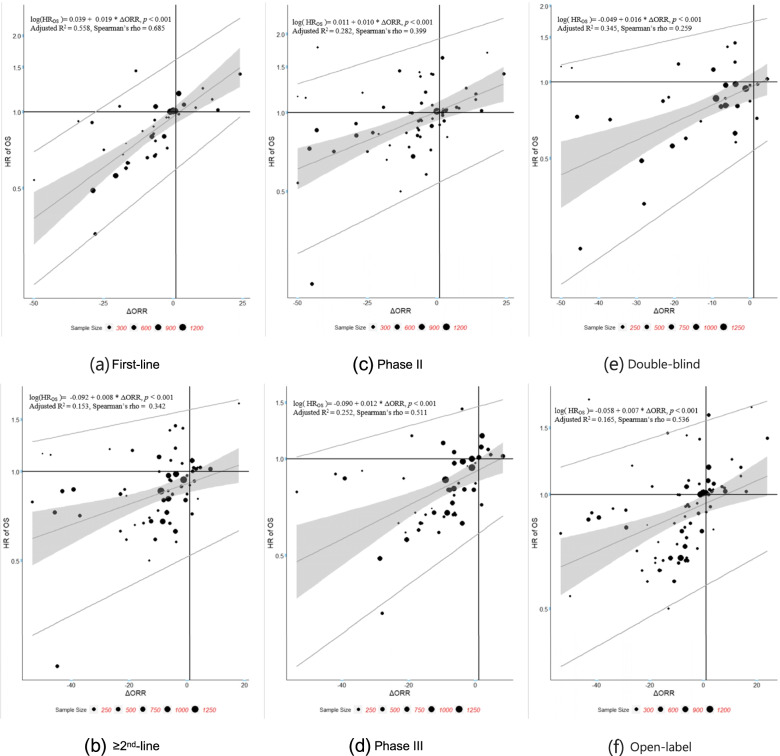
Plots of ΔORR vs. HR of OS by study sample size regression line. Each trial is represented by a bubble whose radius is proportional to the trial sample size

**Fig. 4:**
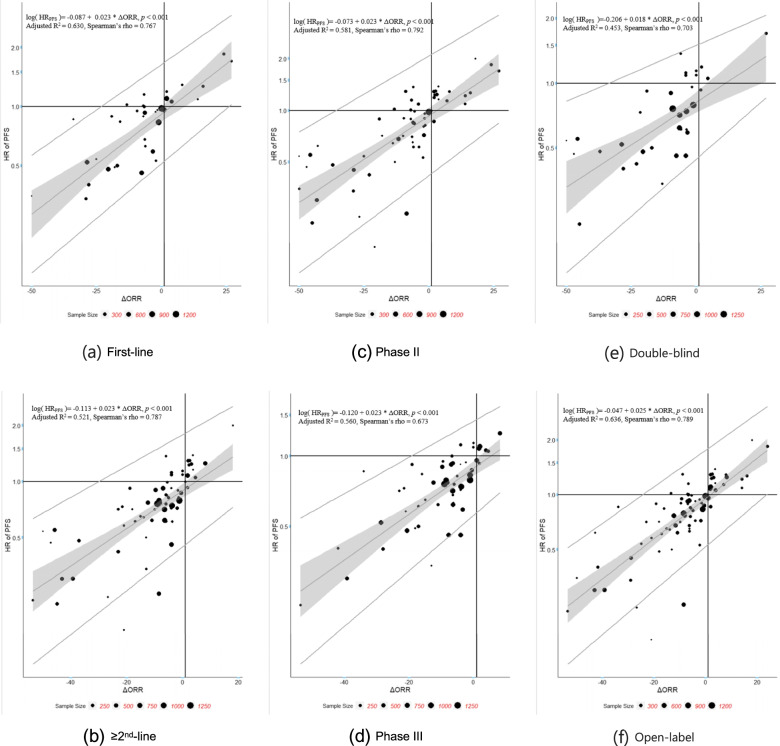
Plots of ΔORR vs. HR of PFS by study sample size regression line. Each trial is represented by a bubble whose radius is proportional to the trial sample size

**Fig. 5:**
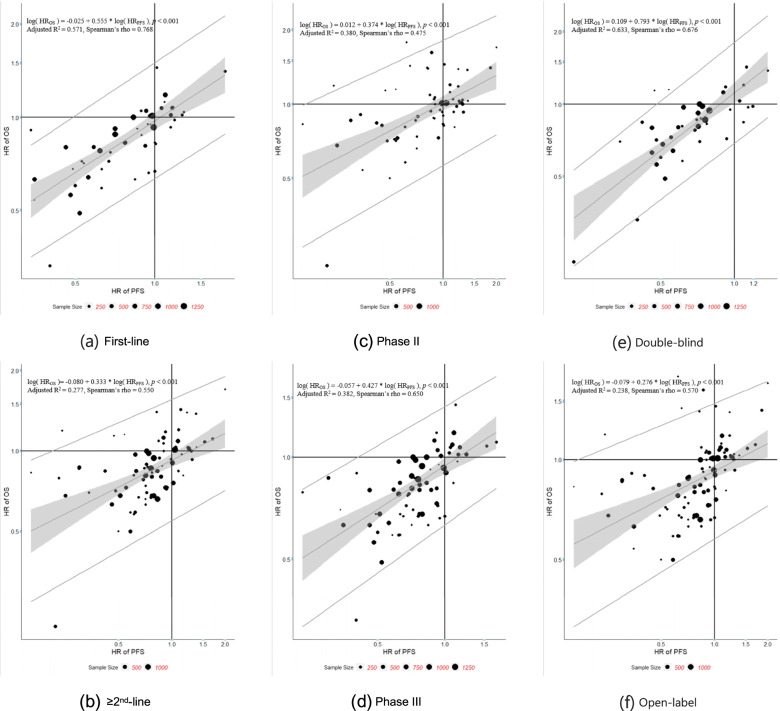
Plots of HR of PFS vs. HR of OS by study sample size regression line. Each trial is represented by a bubble whose radius is proportional to the trial sample size

Sensitivity analyses were performed to evaluate potential heterogeneity in the correlations caused by different trial settings such as line of therapy, phase of trial, masking and therapy type. The results are shown in Tables 6, 7, and 8**,** Figs. 3, 4, and 5. The scatter plots and fitted weighted linear regression lines of ΔORR vs. HR of OS, ΔORR vs. HR of PFS, and HR of PFS vs. HR of OS in different trial settings are shown in Figs. 3, 4, and 5.

**Table 6:** Summary of arm-level sensitivity analysis (Spearman correlation)

Sensitivity Analysis	No. of arms	r_**s**_	95%CI
***ORR ~ median OS***			
**Line of therapy**			
First-line	88	0.636	0.492, 0.746
≥ 2nd-line	146	0.611	0.498, 0.703
**Phase of trial**			
Phase II	141	0.703	0.609, 0.762
Phase III	111	0.660	0.541, 0.754
**Masking**			
Double-blind	66	0.629	0.456, 0.756
Open-label	186	0.710	0.631, 0.775
Therapy*			
Targeted therapy-based	123	0.749	0.660, 0.818
Immunotherapy -based	54	0.449	0.207, 0.640
Chemotherapy-based	65	0.706	0.558, 0.810
**Overall**	254	0.700	0.631,0.758
***ORR ~ median PFS***			
**Line of therapy**			
First-line	93	0.699	0.578, 0.790
≥ 2nd-line	153	0.735	0.653, 0.801
**Phase of trial**			
Phase II	153	0.825	0.768, 0.870
Phase III	117	0.790	0.711, 0.850
**Masking**			
Double-blind	76	0.888	0.828, 0.927
Open-label	193	0.796	0.738, 0.842
Therapy*			
Targeted therapy-based	132	0.823	0.759, 0.871
Immunotherapy -based	58	0.793	0.673, 0.873
Chemotherapy-based	68	0.783	0.669, 0.861
**Overall**	271	0.831	0.790,0.864
***median PFS ~ median OS***			
**Line of therapy**			
First-line	109	0.832	0.763, 0.882
≥ 2nd-line	177	0.599	0.495, 0.686
**Phase of trial**			
Phase II	143	0.798	0.730, 0.851
Phase III	154	0.705	0.616, 0.777
**Masking**			
Double-blind	83	0.727	0.606, 0.815
Open-label	221	0.755	0.692, 0.807
Therapy*			
Targeted therapy-based	132	0.756	0.672, 0.821
Immunotherapy -based	76	0.656	0.506, 0.768
Chemotherapy-based	82	0.786	0.687, 0.857
**Overall**	306	0.755	0.702,0.799

Targeted therapy-based: including targeted therapy with or without placebo/normal care

Immunotherapy: including immunotherapy with or without placebo/normal care

Chemotherapy-based: including conventional chemotherapy with or without placebo/normal care

**Table 7:** Summary of trial-level sensitivity analysis (Spearman correlation)

Sensitivity Analysis	No. of trials	r_**s**_	95%CI
***ΔORR ~ HR of OS***			
**Line of therapy**			
First-line	39	0.685	0.471, 0.822
≥ 2nd-line	64	0.342	0.103, 0.544
**Phase of trial**			
Phase II	60	0.399	0.163, 0.591
Phase III	52	0.511	0.277, 0.688
**Masking**			
Double-blind	35	0.259	−0.081, 0.545
Open-label	76	0.536	0.354, 0.680
**Overall**	112	0.462	0.303, 0.597
***ΔORR ~ HR of PFS***			
**Line of therapy**			
First-line	36	0.767	0.587, 0.875
≥ 2nd-line	68	0.787	0.675, 0.863
**Phase of trial**			
Phase II	60	0.792	0.674, 0.870
Phase III	54	0.673	0.495, 0.797
**Masking**			
Double-blind	36	0.703	0.487, 0.838
Open-label	77	0.789	0.672, 0.852
**Overall**	114	0.764	0.686, 0.861
***HR of PFS ~ HR of OS***			
**Line of therapy**			
First-line	48	0.768	0.621, 0.863
≥ 2nd-line	81	0.550	0.377, 0.686
**Phase of trial**			
Phase II	62	0.475	0.258, 0.647
Phase III	72	0.650	0.492, 0.766
**Masking**			
Double-blind	42	0.676	0.469, 0.813
Open-label	95	0.570	0.417, 0.692
**Overall**	138	0.584	0.462, 0.684

**Table 8:** Summary of trial-level sensitivity analysis (Linear regression)

Sensitivity Analysis	No. of trials	Slope estimate	95%CI	R^**2**^	Adjusted R^**2**^	Threshold
***ΔORR ~ HR of OS***^***a***^						
**Line of therapy**						
First-line	39	0.019	0.013, 0.024	0.570	0.558	−28.01%
≥ 2nd-line	64	0.008	0.004, 0.012	0.166	0.153	——^b^
**Phase of trial**						
Phase II	60	0.010	0.406, 0.014	0.294	0.282	——^b^
Phase III	52	0.012	0.007, 0.018	0.267	0.252	——^b^
**Masking**						
Double-blind	35	0.016	0.009, 0.023	0.365	0.345	——^b^
Open-label	76	0.007	0.004, 0.011	0.176	0.165	——^b^
**Overall**	112	0.011	0.007, 0.014	0.261	0.254	——^b^
***ΔORR ~ HR of PFS***^***a***^						
**Line of therapy**						
First-line	36	0.023	0.018, 0.029	0.641	0.630	−23.25%
≥ 2nd-line	68	0.023	0.018, 0.029	0.528	0.521	−29.95%
**Phase of trial**						
Phase II	60	0.023	0.018, 0.028	0.588	0.581	−35.82%
Phase III	54	0.023	0.017, 0.028	0.568	0.560	−19.28%
**Masking**						
Double-blind	36	0.018	0.012, 0.025	0.468	0.453	−33.76%
Open-label	77	0.025	0.021, 0.030	0.640	0.636	−24.42%
**Overall**	114	0.023	0.019, 0.027	0.578	0.574	−26.30%
***HR of PFS***^***a***^ ***~ HR of OS***^***a***^						
**Line of therapy**						
First-line	48	0.555	0.419, 0.691	0.580	0.571	0.49
≥ 2nd-line	81	0.333	0.217, 0.449	0.286	0.277	——^b^
**Phase of trial**						
Phase II	62	0.374	0.255, 0.492	0.390	0.380	——^b^
Phase III	72	0.427	0.302, 0.553	0.390	0.382	0.41
**Masking**						
Double-blind	42	0.793	0.610, 0.977	0.642	0.633	0.48
Open-label	95	0.276	0.178, 0.374	0.246	0.238	——^b^
**Overall**	138	0.403	0.313, 0.494	0.360	0.355	——^b^

^a^Natural log hazard ratio in the analysis

^b^Only the upper limit of r_s_ is more than 0.7, the STE will be given

**Fig. 6:**
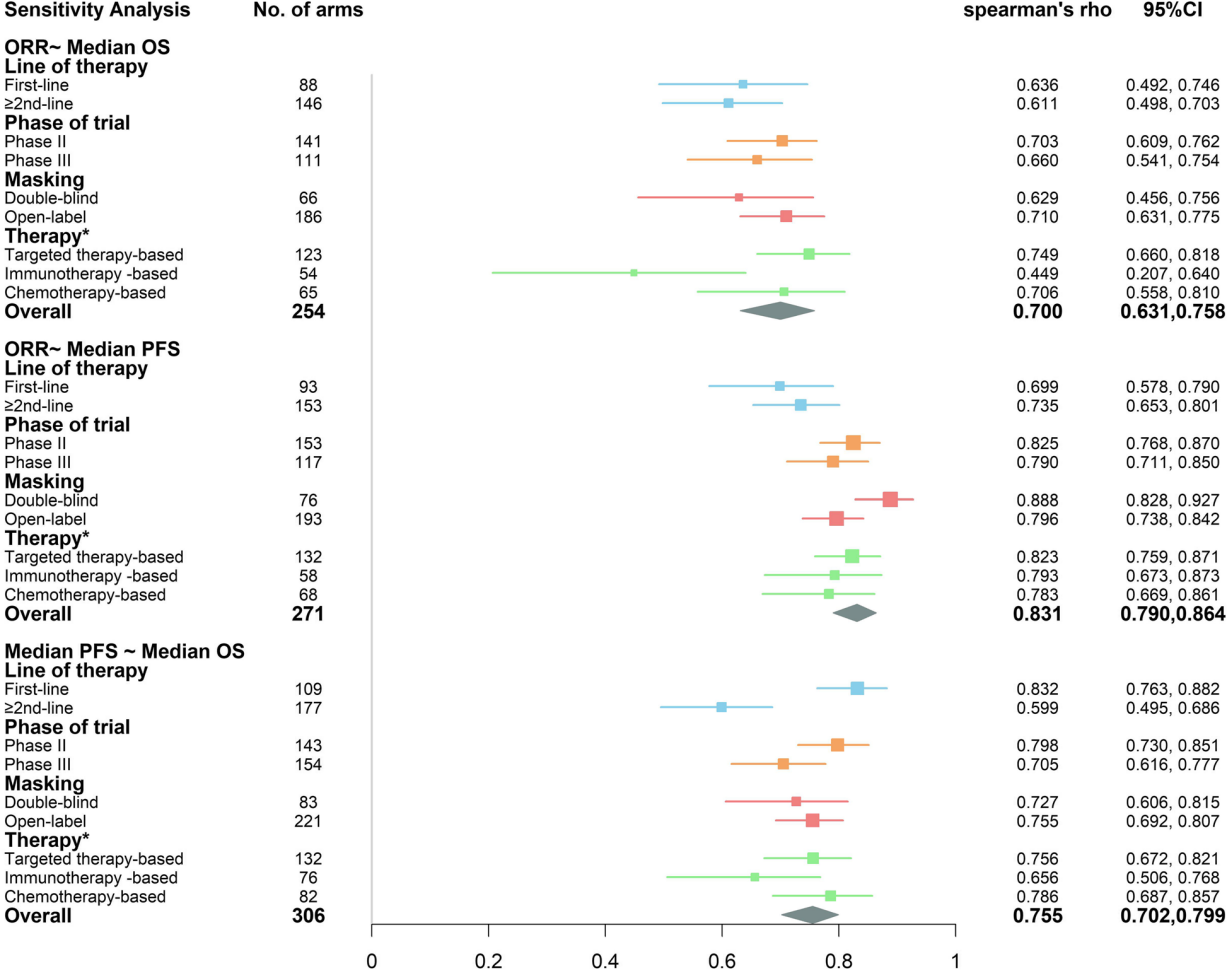
Forest plot of arm-level sensitivity analysis. The box size is based on the precision of Spearman’s rho

From the arm-level sensitivity analysis (Fig. 6), we can find that 1) ORR is more relevant with median OS in first-line, phase II, open-label, targeted-therapy based trials; 2) ORR is more relevant with median PFS in ≥2nd-line, phase II, double-blind, targeted-therapy based trials. 3) PFS is more relevant with median OS in first-line, phase II, open-label, chemotherapy-based trials. Correlations in all subgroups are statistically significant. It is worth mentioning that, in targeted therapy-based trials, the association strengths between ORR and median OS, between ORR and median PFS, and between median PFS and median OS are all at the high positive level. On the contrary, the association strengths in immunotherapy-based trials are much weaker, except for the ORR vs. median PFS pair. We may conclude that at the arm level, surrogate endpoints show the best appropriation in targeted therapy-based trials among all three therapy types.

**Fig. 7:**
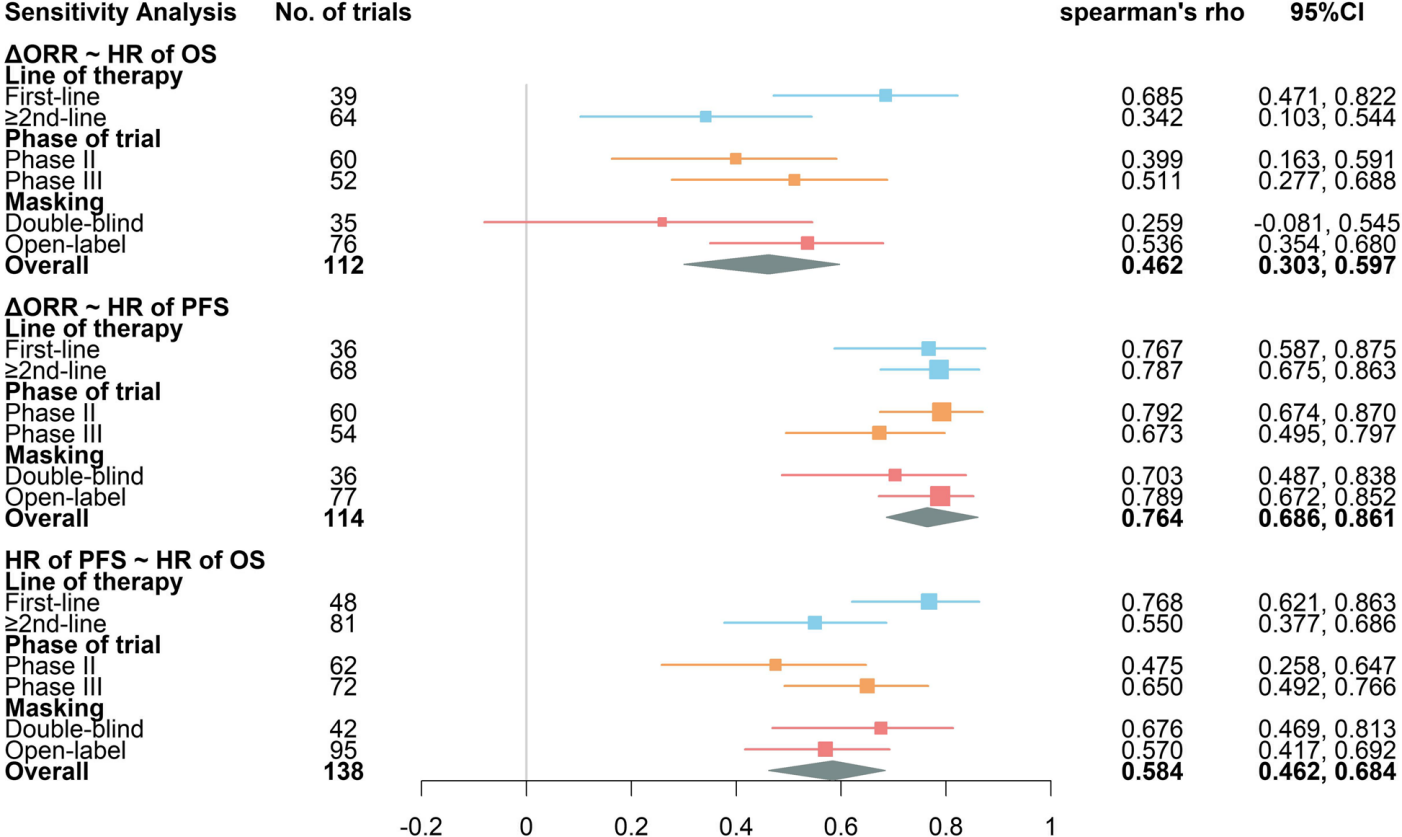
Forest plot of trial-level sensitivity analysis (Spearman correlation). The box size is based on the precision of Spearman’s rho

From the trial-level sensitivity analysis (Fig. 7), we can find that associations between ΔORR and HR of OS are not satisfactory in most subgroups except in first-line therapy trials. The association between ΔORR and HR of PFS is the strongest among all three pairs, with all r_s_ estimated above 0.65 and upper limit of r_s_ estimated above 0.7. For the pair of HR of PFS vs. HR of OS, high positive correlation only exists in first-line therapy trials.

Table 8 reveals the weighted (based on sample size) linear regression coefficient β of surrogate endpoints to true endpoints in each subgroup. All models using ΔORR to predict HR of OS have R^2^ < 0.3 except for that in first-line therapy and double-blind trials, which may lead to the result that there are negligible correlations between ΔORR and HR of OS at trial level_._ In addition, for both ΔORR as a surrogacy of HR of PFS and HR of PFS as a surrogacy of HR of OS, the associations in the subgroup of first-line therapy is much stronger than those in the subgroup of ≥2nd-line therapy, indicating that it may be more reliable and reasonable to use surrogacy endpoints in first-line NSCLC therapy.

## Discussion

All analyses on the arm-level, including those in each subgroup, are all statistically significant. The association between ORR and PFS, and that between PFS and OS, are stronger than the association between ORR and OS, which may indicate a pass-on effect of ORR to PFS to OS.

We can find that the correlation strength sequence is the same at both the arm level and the trial level, with the pair of PFS vs. OS, the pair of ORR vs. PFS, and the pair of ORR vs. OS listed in a descending order. Meanwhile, the value of correlation strength is smaller at the trial level than that at the arm level. This conclusion agrees with Nie [29], Bira [30] and Ritchie [31]. It is worth noting that, one possible reason for the high correlation at arm level may be that the endpoints are naturally dependent by definition. The dependence structure between endpoints, especially for PFS and OS, should be taken into consideration [32]. To explore this, we calculated the mean (0.381) of all arms’ median PFS/median OS, which is much smaller than the arm-level correlation (0.755). So we conclude that the arm-level correlation are rather driven by a real association between PFS and OS than by the dependence introduced because of the definition.

### The surrogacy of ORR for OS

Although ORR has a high positive correlation with OS at arm level, we don’t consider it as an appropriate surrogacy for OS because of its poor performance (low positive correlation, adjusted R^2^ < 0.3) at trial level, as what Gideon [10] has found in advanced NSCLC from both trial level and patient level. However, the additional analysis indicates that when it is limited to trials with first-line therapy, the trial-level association estimates (Spearman’s rho, regression coefficients and adjusted R^2^_)_ increases significantly. Hence, ORR may be used as a surrogate endpoint for OS only in first-line therapy trials.

### The surrogacy of ORR for PFS

At trial level, ΔORR can explain 58% of the variability of HR of PFS. Combining with its good behaviour at arm level, it implies that ORR may be an appropriate surrogacy for PFS, especially in first-line, open-label trials. The fine surrogacy of ORR for PFS to some extent comes from the definition of those two indices. Notably, it seems that ORR works better as surrogacy for PFS in both immunotherapy and targeted therapy than that in chemotherapy.

### The surrogacy of PFS for OS

We can conclude from this analysis that PFS may be at most a modest surrogate endpoint for OS because of its performance at both arm level and trial level, consistent with Buyse’s [15] finding in advanced NSCLC patients and Fiteni‘s [33] finding in locally advanced lung cancer. However, Laporte [14] found that R^2^ were low in advanced NSCLC at patient-level. We also find that the association between PFS and OS is not the same across various therapy types, with stronger association in targeted therapy than in immunotherapy, and both of which are weaker compared to that in chemotherapy. One possible explanation for this finding is that crossover designs are often implemented in immunotherapy and targeted therapy, as mentioned in many researches [34], because of their outstanding early efficacy and ethical consideration, which would make the effect of treatment smaller. Besides, the correlation between PFS and OS is stronger in first-line therapy trials, which was also found by Foster [35] in extensive-stage small cell lung cancer.

There are several limitations in our study that need to be acknowledged. First, meta-analysis can only drop a hint on the association between arm-level and trial-level parameters, while true causation should be established only with an analysis of patient-level data [36]. Second, due to the unavailability of extracting same summary statistics for all trials, such as the OR of ORR on the trial-level, we manually calculated ΔORR using the reported ORR of each arm instead, which may not reflect the true treatment effect of ORR.

At last, the idea can be considered in future studies that dividing all trials into a training part and a validating part, to examine more thoroughly the prediction ability of surrogate endpoints.

## Conclusion

In conclusion, the findings of this meta-analysis of prospective randomized trials demonstrate that ORR is proved to be lack of validity for the surrogacy of OS, except in first-line therapy trials. Conversely, it may be an appropriate surrogate endpoint for PFS across different trial settings (e.g. line of therapy, phase of trial, masking and therapy type), considering the strong and robust correlations at both arm level and trial level. However, as a traditional surrogate endpoint, PFS may be considered at most a modest surrogacy for OS in the setting of phase II/III clinical trials of targeted therapy and immunotherapy for NSCLC patients (especially in first-line therapy trials). Besides, we must admit that to evaluate the true surrogacy, further evaluation of patient-level data should be carried out.

## Supplementary Information

**Additional file 1.** Cochrane Library Search Strategy (May 2021).

**Additional file 2.** Characteristics of Included 136 studies.

## Data Availability

The datasets used and/or analysed during the current study are available from the corresponding author on reasonable request.

## References

[1] Steven A, Fisher SA, Robinson BW (2016). Immunotherapy for lung cancer. Respirology (Carlton, Vic.).

[2] Osmani L (2018). Current WHO guidelines and the critical role of immunohistochemical markers in the subclassification of non-small cell lung carcinoma (NSCLC): moving from targeted therapy to immunotherapy. Semin Cancer Biol.

[3] Naylor EC, Desani JK, Chung PK (2016). Targeted therapy and immunotherapy for lung Cancer. Surg Oncol Clin N Am.

[4] Dermani FK (2019). PD-1/PD-L1 immune checkpoint: potential target for cancer therapy. J Cell Physiol.

[5] Reck M (2016). Pembrolizumab versus chemotherapy for PD-L1-positive non-small-cell lung Cancer. N Engl J Med.

[6] Mauguen A (2013). Surrogate endpoints for overall survival in chemotherapy and radiotherapy trials in operable and locally advanced lung cancer: a re-analysis of meta-analyses of individual patients’ data. Lancet Oncol.

[7] Buyse M (2016). Statistical evaluation of surrogate endpoints with examples from cancer clinical trials. Biometrical J Biometrische Zeitschrift.

[8] Blajman C (1999). A prospective, randomized phase III trial comparing combination chemotherapy with cyclophosphamide, doxorubicin, and 5-fluorouracil with vinorelbine plus doxorubicin in the treatment of advanced breast carcinoma. Cancer.

[9] Johnson KR (2006). Response rate or time to progression as predictors of survival in trials of metastatic colorectal cancer or non-small-cell lung cancer: a meta-analysis. Lancet Oncol.

[10] Blumenthal GM (2015). Overall response rate, progression-free survival, and overall survival with targeted and standard therapies in advanced non-small-cell lung cancer: US Food and Drug Administration trial-level and patient-level analyses. J Clin Oncol.

[11] Hotta K (2007). Relationship between response and survival in more than 50,000 patients with advanced non-small cell lung cancer treated with systemic chemotherapy in 143 phase III trials. J Thoracic Oncol.

[12] Hayashi H (2013). Postprogression survival in patients with advanced non-small-cell lung cancer who receive second-line or third-line chemotherapy. Clin Lung Cancer.

[13] Soria JC, Massard C, Le Chevalier T (2010). Should progression-free survival be the primary measure of efficacy for advanced NSCLC therapy?. Ann Oncol.

[14] Laporte S, et al. Prediction of survival benefits from progression-free survival benefits in advanced non-small-cell lung cancer: evidence from a meta-analysis of 2334 patients from 5 randomised trials. BMJ Open. 2013;3(3):e001802. 10.1136/bmjopen-2012-001802 PMC3612819 23485717

[15] Buyse ME (2008). Prediction of survival benefits from progression-free survival in patients with advanced non small cell lung cancer: evidence from a pooled analysis of 2,838 patients randomized in 7 trials. J Clin Oncol.

[16] Moher D (2009). Preferred reporting items for systematic reviews and meta-analyses: the PRISMA statement. Plos Med.

[17] Prentice RL (1989). Surrogate endpoints in clinical trials: definition and operational criteria. Stat Med.

[18] Burzykowski T (2001). Validation of surrogate end points in multiple randomized clinical trials with failure time end points. J R Stat Soc C.

[19] Buyse M (2008). Individual- and trial-level surrogacy in colorectal cancer. Stat Methods Med Res.

[20] Zhang J (2019). Endpoint surrogacy in oncological randomized controlled trials with immunotherapies: a systematic review of trial-level and arm-level meta-analyses. Ann Transl Med.

[21] Li YF (2022). Surrogate endpoints for overall survival in immune-oncology trials of advanced gastro-esophageal carcinoma. World J Oncol.

[22] Kaufman HL (2018). Evaluation of classical clinical endpoints as surrogates for overall survival in patients treated with immune checkpoint blockers: a systematic review and meta-analysis. J Cancer Res Clin Oncol.

[23] Goring S (2022). Correlations between objective response rate and survival-based endpoints in first-line advanced non-small cell lung Cancer: a systematic review and meta-analysis. Lung Cancer.

[24] Delea TE (2012). Association between treatment effects on disease progression end points and overall survival in clinical studies of patients with metastatic renal cell carcinoma. Br J Cancer.

[25] Burzykowski T (2008). Evaluation of tumor response, disease control, progression-free survival, and time to progression as potential surrogate end points in metastatic breast cancer. J Clin Oncol.

[26] Hackshaw A (2005). Surrogate markers and survival in women receiving first-line combination anthracycline chemotherapy for advanced breast cancer. Br J Cancer.

[27] Prasad V (2015). The strength of association between surrogate end points and survival in oncology: a systematic review of trial-level Meta-analyses. JAMA Intern Med.

[28] Burzykowski T, Buyse M (2006). Surrogate threshold effect: an alternative measure for meta-analytic surrogate endpoint validation. Pharm Stat.

[29] Nie R-C (2019). Evaluation of objective response, disease control and progression-free survival as surrogate end-points for overall survival in anti-programmed death-1 and anti-programmed death ligand 1 trials. Eur J Cancer (Oxford, England: 1990).

[30] Bria E (2015). Progression-free survival as primary endpoint in randomized clinical trials of targeted agents for advanced renal cell carcinoma. Correlation with overall survival, benchmarking and power analysis. Crit Rev Oncol Hematol.

[31] Ritchie G (2018). Defining the Most appropriate primary end point in phase 2 trials of immune checkpoint inhibitors for advanced solid cancers: a systematic review and Meta-analysis. JAMA Oncol.

[32] Fleischer F, Gaschler-Markefski B, Bluhmki E (2009). A statistical model for the dependence between progression-free survival and overall survival. Stat Med.

[33] Fiteni F, Westeel V, Bonnetain F (2017). Surrogate endpoints for overall survival in lung cancer trials: a review. Expert Rev Anticancer Ther.

[34] Hashim M, et al. Do surrogate endpoints better correlate with overall survival in studies that did not allow for crossover or reported balanced postprogression treatments? An application in advanced non-small cell lung cancer. Value Health. 2018;21(1):9–17. 10.1016/j.jval.2017.07.011 29304946

[35] Foster NR (2015). Multitrial evaluation of progression-free survival as a surrogate end point for overall survival in first-line extensive-stage small-cell lung Cancer. J Thoracic Oncol.

[36] Piedbois P, Buyse M (2004). Meta-analyses based on abstracted data: a step in the right direction, but only a first step. J Clin Oncol.

