# Supplementary material for: Validating ORR and PFS as surrogate endpoints in phase II and III clinical trials for NSCLC patients: difference exists in the strength of surrogacy in various trial settings
Source: BMC Cancer. 2022 Sep 29;22:1022. doi: 10.1186/s12885-022-10046-z (PMC9520950; doi:10.1186/s12885-022-10046-z)
Supplement: Supplementary file 1 — Additional file 1. Cochrane Library Search Strategy (May 2021). [file 12885_2022_10046_MOESM1_ESM.docx]

**Additional file 1: Cochrane Library Search Strategy (May 2021)**

#1 MeSH descriptor: [Carcinoma, Non-Small-Cell Lung] explode all trees

#2 (nsclc or NSCLC or 'non small cell lung cancer'):ti,ab,kw

#3 (os or OS or 'overall survival'):ti,ab,kw

#4 MeSH descriptor: [Progression-Free Survival] explode all trees

#5 (pfs or PFS or 'progression free survival'):ti,ab,kw

#6 (orr or ORR or 'objective response rate' or ‘response rate’):ti,ab,kw

#7 MeSH descriptor: [Immunotherapy] explode all trees

#8 (immunotherapy or immun* or *umab):ti,ab,kw

#9 MeSH descriptor: [Molecular Targeted Therapy] explode all trees

#10 ('targeted therapy' or *tinib):ti,ab,kw

#11 #1 or #2

#12 #4 or #5

#13 #7 or #8

#14 #9 or #10

#15 #11 and #3 and (#12 or #6) and (#13 or #14)
