# Supplementary material for: Validating ORR and PFS as surrogate endpoints in phase II and III clinical trials for NSCLC patients: difference exists in the strength of surrogacy in various trial settings
Source: BMC Cancer. 2022 Sep 29;22:1022. doi: 10.1186/s12885-022-10046-z (PMC9520950; doi:10.1186/s12885-022-10046-z)
Supplement: Supplementary file 2 — Additional file 2. Characteristics of Included 136 studies. [file 12885_2022_10046_MOESM2_ESM.docx]

**Additional file 2: Characteristics of Included 136 studies**

| **ID** | **Reference** | **Stage** | **Line** | **Treatment type&N** | **N** | **Phase** | **Masking** | **NCT number** |
| --- | --- | --- | --- | --- | --- | --- | --- | --- |
| 1 | Zhou, C., et al. (2020) | IIIB or IV | second-line or later | pyrotinib 60 | 60 | II | Open-label | NCT02834936 |
| 2* | Zhao, S., et al. (2020) | IIIB or IV | all | erlotinib 85 gefitinib 86 | 171 | II | Open-label | NCT01955421 |
| 3 | Zhang, X., et al. (2020) | IIIB or IIIC or IV | third-line or later | apatinib 30 | 37 | II | Open-label | NCT03652857 |
| 4 | Ramalingam, S. S., et al. (2020) | advanced | first-line | osimertinib 279 gefitinib/erlotinib 277 | 556 | III | Double-blind | NCT02296125 |
| 5 | Papadimitrakopoulou, V. A., et al. (2020) | advanced | second-line or later | osimertinib 279 pemetrexed+carboplatin/cisplatin 136 | 415 | III | Open-label | NCT02151981 |
| 6 | Nishio, M., et al. (2020) | IV | third-line or later | ceritinib 124 | 124 | II | Open-label | NCT01685138 |
| 7 | Huber, R. M., et al. (2020) | IV | first-line | brigatinib 90mg 112 brigatinib 180mg 110 | 222 | II | Open-label | NCT02094573 |
| 8 | Goldberg, S. B., et al. (2020) | advanced | first-line | afatinib+cetuximab 83 afatinib 85 | 168 | II/III | Open-label | NCT02438722 |
| 9 | Cho, J. H., et al. (2020) | metastatic | all | osimertinib 37 | 30 | II | Open-label | NCT03424759 |
| 10 | Reckamp, K. L., et al. (2019) | advanced | all | cabozantinib+erlotinib 37 | 37 | II | Open-label | NCT01866410 |
| 11 | Moro-Sibilot, D., et al. (2019) | advanced | all | MET>6 copies cohort 25 MET-mutated cohort 28 ROS-1-translocated cohort 37 | 90 | II | Open-label | NCT02034981 |
| 12 | Melosky, B., et al. (2019) | advanced | all | selumetinib 75mg twice a day 20 selumetinib 75mg once a day 21 no selumetinib 21 | 62 | II | Open-label | NCT02337530 |
| 13 | Kelly, R. J., et al. (2019) | IIIB or IV | all | ASP8273 267 erlotinib/gefitinib 263 | 530 | III | Open-label | NCT02588261 |
| 14 | Jiao, L., et al. (2019) | III or IV | third-line or later | gefitinib/erlotinib/icotinib+CHM 226 gefitinib/erlotinib/icotinib+placebo 225 | 451 | III | Double-blind | NCT01745302 |
| 15 | Wu, Y. L., et al. (2018) | advanced | first-line | crizotinib 104 chemotherapy 103 | 207 | III | Open-label | NCT01639001 |
| 16 | Soria, J. C., et al. (2018) | advanced | first-line | osimertinib 279 standard EGFR-TKIs 277 | 556 | III | Double-blind | NCT0229612 |
| 17 | Scagliotti, G. V., et al. (2018) | advanced | second-line or later | erlotinib +tivantinib 56 erlotinib+placebo 53 | 109 | III | Double-blind | NCT01244191 |
| 18 | Mok, T. S., et al. (2018) | IIIB/IV | first-line | dacomitinib 227  gefitinib 225 . | 452 | III | Open-label | NCT01774721 |
| 19 | Lu, S., et al. (2018) | advanced | third-line or later | fruquintinib 61 placebo 30 | 91 | II | Double-blind | NCT02590965 |
| 20 | Han, B., et al. (2018) | IIIB/IV | third-line or later | anlotinib 296 placebo 143 | 439 | III | Double-blind | NCT02388919 |
| 21 | Garon, E. B., et al. (2018) | IIIB/IV | second-line or later | erlotinib+fulvestrant 73 erlotinib alone 33 | 106 | II | Open-label | NCT00100854 |
| 22 | Soria, J. C., et al. (2017) | advanced | first-line | ceritinib 189 chemotherapy 187 | 376 | III | Open-label | NCT01828099 |
| 23 | Soria, J. C., et al. (2017) | advanced | second-line | selumetinib+docetaxel60 85 selumetinib+docetaxel75 84 placebo+docetaxe75l 43 | 212 | II | Double-blind | NCT01750281 |
| 24 | Lee, Y., et al. (2017) | advanced | second-line or later | afatinib+simvastatin 36 afatinib 32 | 68 | II | Open-label | NCT01156545 |
| 25 | Jänne, P. A., et al. (2017) | IIIB/IV | second-line | selumetinib+docetaxel 254 docetaxel 256 | 510 | III | Double-blind | NCT01933932 |
| 26 | Ciuleanu, T. E., et al. (2017) | IIIB/IV | second-line or later | linsitinib/erlotinib 102 placebo/erlotini 103 | 205 | II | Double-blind | NCT01186861 |
| 27 | Yang, J. C., et al. (2016) | advanced | first-line | pemetrexed+cisplatin+gefitinib 118 gefitinib 118 | 236 | III | Open-label | NCT01017874 |
| 28 | Smit, E. F., et al. (2016) | IIIB/IV | second-line | erlotinib 300mg 160 erlotinib 150mg 155 | 315 | III | Double-blind | NCT01183858 |
| 29* | Schuler, M., et al. (2016) | IIIB/IV | first-line | LUX-Lung 3 afatinib 230 cisplatin-pemetrexed 115 LUX-Lung 6: afatinib 242 cisplatin-pemetrexed 122 | 709 | III | Open-label | NCT00949650  NCT01121393 |
| 30 | Park, K., et al. (2016) | not said | first-line | erlotinib 359 | 359 | II | Open-label | NCT01310036 |
| 31 | Kim, Y. S., et al. (2016) | IIIB/IV | second-line | pemetrexed 47  gefitinib 48 | 95 | II | Open-label | NCT01783834 |
| 32 | Cheng, Y., et al. (2016) | IV or recurrent | first-line | pemetrexed+gefitinib 129 gefitinib monotherapy 66 | 195 | II | Open-label | NCT01469000 |
| 33 | Zhong, W., et al. (2015) | IIIA-N2 | first-line or second-line | neoadjuvant erlotinib 12 gemcitabine/carboplatin 12 | 24 | II | Open-label | NCT00600587 |
| 34 | Yoshioka, H., et al. (2015) | IIIB/IV | second-line or later | placebo+erlotinib 153  tivantinib+erlotinib 154 | 307 | III | Double-blind | NCT01377376 |
| 35 | Wu, Y. L., et al. (2015) | IIIB/IV | first-line | erlotinib 110 gemcitabine 107 | 217 | III | Open-label | NCT01342965 |
| 36* | Soria, J. C., et al. (2015) | IIIB/IV | second-line or later | gefitinib+cisplatin+pemetrexed 133  Placebo+cisplatin+pemetrexed 132 | 265 | III | Double-blind | NCT01544179 |
| 37 | Scagliotti, G., et al. (2015) | IIIB/IV | second-line or later | erlotinib+tivantinib 526 erlotinib+placebo 522 | 1048 | III | Double-blind | NCT01244191 |
| 38 | Choi, Y. J., et al. (2015) | IIIB/IV | first-line | chemotherapy+gefitinib PCG 44  chemotherapy PC 46 | 90 | II | Open-label | NCT01196234 |
| 39 | Bondarenko, I. M., et al. (2015) | IIIB/IV | not said | cisplatin+gemcitabine+axitinib 38 | 38 | II | Open-label | NCT00735904 |
| 40 | Blumenschein, G. R., et al. (2015) | IV | second-line | trametinib 86 docetaxel 43 | 129 | II | Open-label | NCT01362296 |
| 41 | Zhou, Q., et al. (2014) | IIIB/IV | second-line | pemetrexed 76 gefitinib 81 | 157 | II | Open-label | NCT00891579 |
| 42 | Solomon, B. J., et al. (2014) | advanced | first-line | crizotinib 172 chemotherapy 171 | 343 | III | Open-label | NCT01154140 |
| 43 | Karavasilis, V., et al. (2014) | IV | first-line | erlotinib+docetaxel 55 gefitinib+docetaxel 55 | 110 | II | Open-label | NCT00783471 |
| 44* | Gitlitz, B. J., et al. (2014) | IIIB/IV | second-line or later | apricoxib+erlotinib 75 placebo+erlotinib 39 | 114 | II | Open-label | NCT00652340 |
| 45 | Dittrich, C., et al. (2014) | III-IV | second-line | pemetrexed 83 pemetrexed+erlotinib 76 | 159 | II | Open-label | NCT00447057 |
| 46 | Belani, C. P., et al. (2014) | IIIB/IV | first-line | axitinib bid continuously+pemetrexed/cisplatin 55 axitinib in a modified-dOSing schedule+pemetrexed/cisplatin 58 pemetrexed/cisplatin alone 57 | 170 | II | Open-label | NCT00768755 |
| 47 | Auliac, J. B., et al. (2014) | IIIB/IV | second-line or later | docetaxel+erlotinib 73 docetaxel 74 | 147 | II | Open-label | NCT01350817 |
| 48 | Katakami, N., et al. (2013) | IIIB/IV | second-line or later | afatinib monotherapy 62 | 62 | II | Open-label | NCT00711594 |
| 49 | Karampeazis, A., et al. (2013) | IIIB/IV | second-line or later | pemetrexed 166 erlotinib 166 | 332 | III | Open-label | NCT00440414 |
| 50* | Jänne, P. A., et al. (2013) | IIIB/IV | second-line or later | selumetinib+docetaxel 43 MG1 24 MG2 19 Placebo+docetaxel 40 MG1 23 MG2 17 MG:mutation groups MG1:KRAS G12C or G12V MG2:all KRAS mutations other than G12C or G12V | 83 | II | Double-blind | NCT00890825 |
| 51 | Groen, H. J. M., et al. (2013) | IIIB/IV | second-line | sunitinib+erlotinib 65 placebo+erlotinib 67 | 132 | II | Double-blind | NCT00265317 |
| 52* | Aerts, J. G., et al. (2013) | Ib/IIIb/IV | second-line or later | monotherapy 115  combination 116 | 231 | II | Open-label | NCT00835471 |
| 53 | Zhang, L., et al. (2012) | Ib/IIIb/IV | second-line or later | gefitinib 147 placebo 148 | 295 | III | Double-blind | NCT00770588 |
| 54 | Scagliotti, G. V., et al. (2012) | IIIB/IV | second-line or later | tivantinib+erlotinib 58 placebo+erlotinib 59 | 117 | III | Double-blind | NCT01244191 |
| 55 | Chen, Y. M., et al. (2012) | IIIB/IV | first-line | erlotinib 57 vinorelbine 56 | 113 | II | Open-label | NCT01196078 |
| 56 | Bauman, J. E., et al. (2012) | IIIB-IV | first-line | imatinib and paclitaxel 34 | 34 | II | Open-label | NCT01011075 |
| 57 | Ramalingam, S. S., et al. (2011) | IIIB/IV | second-line or third-line | erlotinib+Placebo 57  erlotinib+R1507 weekly 57  erlotinib+R1507 every 3 weeks 57 | 171 | II | Double-blind | NCT00760929 |
| 58 | Natale, R. B., et al. (2011) | IIIB/IV | second-line or later | vandetanib 623 erlotinib 614 | 1237 | III | Double-blind | NCT00364351 |
| 59 | Han, J. Y., et al. (2011) | IIIB/IV | second-line or later | gefitinib+simvastatin 52 gefitinib 54 | 106 | II | Open-label | NCT00452244 |
| 60 | Gaafar, R. M., et al. (2011) | IIIB/IV | second-line or later | placebo 87 gefitinib consolidation 86 | 173 | III | Double-blind | NCT00091156 |
| 61 | Lee, D. H., et al. (2010) | IIIB/IV | second-line or later | gefitinib 81 docetaxel 76 | 157 | III | Open-label | NCT00478049 |
| 62* | Douillard, J. Y., et al. (2010) | advanced | second-line or third-line | gefitinib 729 docetaxel 715 | 1444 | III | Open-label | NCT00076388 |
| 63* | Cappuzzo, F., et al. (2010) | IIIB/IV | second-line | erlotinib 438 placebo 451 | 889 | III | Double-blind | NCT00556712 |
| 64 | Goss, G., et al. (2009) | locally advanced/ metastatic | first-line | gefitinib 100 placebo 101 | 201 | II | Double-blind | NCT00259064 |
| 65 | Simon, G. R., et al. (2008) | IIIB/IV | first-line | docetaxel+ZD1839 44 | 44 | II | Open-label | NCT00231465 |
| 66 | Maruyama, R., et al. (2008) | IIIB-IV | second-line or later | gefitinib 245 docetaxel 244 | 489 | III | Open-label | NCT00252707 |
| 67 | Crinò, L., et al. (2008) | IIIB/IV | first-line | gefitinib 97  vinorelbine 99 | 196 | II | Open-label | NCT00256711 |
| 68 | Park, K., et al. (2016) | IIIB and IV | first-line | afatinib 40mg per day 160 gefitinib 250mg per day 159 | 319 | IIB | Open-label | NCT01466660 |
| 69 | Wu, Y. L., et al. (2018) | IIIB/ IV | first-line | afatinib 217  gemcitabine/cisplatin 110 | 327 | III | Open-label | NCT01121393 |
| 70 | Papadimitrakopoulou, V., et al. (2016) | not said | second-line or later | erlotinib 22 erlotinib+MK-2206 42 MK-2206+AZD6244 75 sorafenib 61 | 200 | II | Open-label | NCT01248247 |
| 71 | Wu, Y. L., et al. (2021) | not said | first-line | pembrolizumab 128 platinum‐based chemotherapy 134 | 262 | III | Open-label | NCT02220894 |
| 72 | Gadgeel, S., et al. (2020) | advanced | first-line | pembrolizumab+pemetrexed+platinum 410 placebo+pemetrexed+platinum  206 | 616 | III | Double-blind | NCT02578680 |
| 73 | Cortot, A. B., et al. (2020) | advanced | second-line or third-line | docetaxel 55  paclitaxel+bevacizumab 111 | 166 | III | Open-label | NCT01763671 |
| 74 | Chen, Y. M., et al. (2020) | IIIB/IV | second-line or later | nivolumab 53 | 53 | II | Open-label | NCT02582125 |
| 75* | Arrieta, O., et al. (2020) | metastatic | second-line | pembrolizumab+docetaxel 40 docetaxel 38 | 78 | II | Open-label | NCT02574598 |
| 76 | Wu, Y. L., et al. (2019) | IIIB or IV | second-line or later | nivolumab 338  docetaxel 166 | 504 | III | Open-label | NCT02613507 |
| 77 | Villaruz, L. C., et al. (2019) | IV | first-line | nab-paclitaxel and carboplatin chemotherapy+necitumuma 54 | 54 | II | Open-label | NCT02392507 |
| 78 | Theelen, W. S. M. E., et al. (2019) | advanced | second-line or later | pembrolizumab+stereotactic body radiotherapy 36 pembrolizumab therapy alone 40 | 76 | II | Open-label | NCT02492568 |
| 79 | Stinchcombe, T. E., et al. (2019) | IV | not said | erlotinib 45 erlotinib+Bevacizumab 43 | 88 | II | Single-blind | NCT01532089 |
| 80 | Sequist, L. V., et al. (2019) | advanced | second-line or later | seribantumab+erlotinib 85 erlotinib 44 | 129 | II | Open-label | NCT02387216 |
| 81 | Reck, M., et al. (2019) | IV | first-line | pembrolizumab 154  chemotherapy 151 | 305 | III | Open-label | NCT02142738 |
| 82 | Patil, P. D., et al. (2019) | IIIB/IV | first-line | docetaxel‐gemcitabine‐bevacizumab 13 | 13 | II | Open-label | NCT00970684 |
| 83 | Levy, B. P., et al. (2019) | IIIB/IV | second-line | pembrolizumab+CC-486 51 pembrolizumab+placebo 49 | 100 | II | Double-blind | NCT02546986 |
| 84* | Herbst, R. S., et al. (2019) | locally advanced or metastatic | second-line or later | pembrolizumab 690 docetaxelb 343 | 1033 | II/III | Open-label | NCT01905657 |
| 85 | von Pawel, J., et al. (2018) | IV | first-line | parsatuzumab+paclitaxel+carboplatin+bevacizumab 98 paclitaxel+carboplatin+bevacizumab 100 | 198 | II | Double-blind | NCT01366131 |
| 86 | Spigel, D. R., et al. (2018) | IIIB/IV | first-line | pemetrexed 48  pemetrexed+bevacizumab 63  pemetrexed+bevacizumab+carboplatin 61 | 172 | II | Open-label | NCT00892710 |
| 87 | Paz-Ares, L., et al. (2018) | IV | first-line | pembrolizumab-combination group 278 placebo-combination group 281 | 559 | III | Double-blind | NCT02775435 |
| 88 | Nie, K., et al. (2018) | IV | third-line | osimertinib 74 docetaxel-bevacizumab 73 | 147 | III | Open-label | NCT02959749 |
| 89 | Nakao, A., et al. (2018) | IIIB/IV | first-line | carboplatin+nab-paclitaxel 39 | 39 | II | Open-label | NCT02027428 |
| 90 | Marrone, K. A., et al. (2018) | IIIB/IV | not said | chemotherapy+metformin 19 chemotherapy alone 6 | 25 | II | Open-label | NCT01578551 |
| 91 | Herbst, R. S., et al. (2018) | IV | first-line | chemotherapy+cetuximab 656 chemotherapy without cetuximab 657 | 1313 | III | Open-label | NCT00946712 |
| 92 | Gridelli, C., et al. (2018) | not said | second-line or later | bevacizumab+standard of care 243 bevacizumab alone 232 | 475 | IIIb | Open-label | NCT01351415 |
| 93 | Gandhi, L., et al. (2018) | metastatic | first-line | pembrolizumab combination 410 placebo combination 206 | 616 | III | Double-blind | NCT02578680 |
| 94 | Engel-Riedel, W., et al. (2018) | IIIB or IV | first-line | BTH1677/bevacizumab/carboplatin/paclitaxel 48 bevacizumab/carboplatin/paclitaxel 23 | 71 | II | Open-label | NCT00874107 |
| 95 | Ciuleanu, T., et al. (2018) | IV | first-line | necitumumab with gemcitabine-cisplatin 261 gemcitabine-cisplatin 215 | 476 | III | Open-label | NCT00981058 |
| 96 | Antonia, S. J., et al. (2017) | III | second-line or later | durvalumab 473 placebo 236 | 709 | III | Double-blind | NCT02125461 |
| 97 | Spigel, D. R., et al. (2017) | IV | first-line | necitumumab With paclitaxel-carboplatin 110  paclitaxel-carboplatin 57 | 167 | II | Open-label | NCT01769391 |
| 98 | Novello, S., et al. (2017) | IV | first-line | cixutumumab+pemetrexed+cisplatin 87 pemetrexed 85 | 172 | II | Open-label | NCT01233452 |
| 99 | Kim, H. R., et al. (2017) | IV | second-line | nimotuzumab+Gefitinib 78  gefitinib 75 | 153 | II | Open-label | NCT01498562 |
| 100 | Gerber, D. E., et al. (2017) | IIIB/IV | first-line | olaratumab+paclitaxel-carboplatin 67  paclitaxel-carboplatin 64 | 131 | II | Open-label | NCT00918203 |
| 101* | Carbone, D. P., et al. (2017) | IV or recurrent | first-line | nivolumab 271 platinum doublet chemotherapy 270 | 541 | III | Open-label | NCT02041533 |
| 102 | Yoh, K., et al. (2016) | IV | second-line | ramucirumab-docetaxel  76 placebo-docetaxel 81 | 157 | II | Double-blind | NCT01703091 |
| 103 | Reck, M., et al. (2016) | IV | first-line | pembrolizumab  154 chemotherapy 151 | 305 | III | Double-blind | NCT02142738 |
| 104 | Zinner, R. G., et al. (2015) | IV | first-line | pemetrexed+carboplatin 182 paclitaxel+carbo platin+bevacizumab 179 | 361 | III | Open-label | NCT00948675 |
| 105 | Thomas, M., et al. (2015) | IIIB/IV | first-line | EB erlotinib+bevacizumab 111 PGB cisplatin/gemcitabine+bevacizumab 113 | 224 | II | Open-label | NCT00536640 |
| 106 | Scagliotti, G. V., et al. (2015) | IIIB/IV or recurrent | second-line or later | figitumumab+erlotinib 293 erlotinib 290 | 583 | III | Open-label | NCT00673049 |
| 107 | Kotsakis, A., et al. (2015) | IIIB/IV | first-line | docetaxel/gemcitabine/bevacizumab 38 DCB docetaxel/cisplatin/bevacizumab 39 | 77 | II | Open-label | NCT00620971 |
| 108 | Dingemans, A. M., et al. (2015) | IV | first-line | carboplatin–paclitaxel–bevacizumab 112 carboplatin–paclitaxel–bevacizumab+Nitroglycerin 111 | 223 | II | Open-label | NCT01171170 |
| 109 | Brahmer, J., et al. (2015) | IIIB/IV | second-line | nivolumab 135  docetaxel 137 | 272 | III | Open-label | NCT00730639 |
| 110 | Borghaei, H., et al. (2015) | IIIB/IV | second-line and third-line | nivolumab 292  docetaxel 290 | 582 | III | Open-label | NCT01673867 |
| 111 | Paz-Ares, L., et al. (2015) | IV | first-line | Necitumumab+pemetrexed and cisplatin 315  Pemetrexed and cisplatin alone 318 | 633 | III | Open-label | NCT00982111 |
| 112 | Twelves, C., et al. (2014) | IIIB/IV | first-line | axitinib+paclitaxel/carboplatin 58  bevacizumab+paclitaxel/carboplatin 60 | 118 | II | Open-label | NCT00600821 |
| 113 | Reck, M., et al. (2014) | IIIB/IV | second-line | docetaxel+nintedanib 655  docetaxel+placebo 659 | 1314 | III | Double-blind | NCT00805194 |
| 114 | Langer, C. J., et al. (2014) | IIIB/IV | first-line | figitumumab+paclitaxel and carboplatin 342  paclitaxel and carboplatin alone 339 | 681 | III | Open-label | NCT00596830 |
| 115 | Garon, E. B., et al. (2014) | IV | second-line | ramucirumab+docetaxe 628  placebo+docetaxel group 625 | 1253 | III | Double-blind | NCT01168973 |
| 116 | Reck, M., et al. (2013) | IIIB/IV | first-line | placebo+carboplatin/paclitaxel 48 tigatuzumab+carboplatin/paclitaxel 49 | 97 | II | Double-blind | NCT00991796 |
| 117 | Patel, J. D., et al. (2013) | IIIB/IV | first-line | pemetrexed+carboplatin+bevacizumab 472 paclitaxel+carboplatin+bevacizumab 467 | 939 | III | Open-label | NCT00762034 |
| 118 | Ciuleanu, T., et al. (2013) | IIIB/IV | first-line | bevacizumab+chemotherapy 61 bevacizumab+erlotinib 63 | 124 | II | Open-label | NCT00531960 |
| 119 | Garon, E. B., et al. (2012) | IV | second-line | ramucirumab+docetaxel group 628  placebo+docetaxel group 625 | 1253 | III | Double-blind | NCT01168973 |
| 120 | Herbst, R. S., et al. (2011) | recurrent or refractory advanced- | second-line or later | erlotinib+placebo control group 317 erlotinib+bevacizumab bevacizumab group 319 | 636 | III | Double-blind | NCT00130728 |
| 121 | Blumenschein, G. R., et al. (2011) | IIIB/IV | not said | armA motesanib 125 61 armB motesanib 75 62 armC motesanib 15 63 | 186 | II | Open-label | NCT00369070 |
| 122* | Planchard, D., et al. (2020) | IIIB/IV | third-line or later | study A TC ≥25% : durvalumab 62 SoC 64 study B TC <25% : durvalumab+tremelimumab 174 SoC 118 durvalumab 117 tremelimumab 60 | 595 | III | Open-label | NCT02352948 |
| 123 | Socinski, M. A., et al. (2018) | IV or recurrent metastatic | first-line | atezolizumab+carboplatin+paclitaxel ACP 402 bevacizumab+carboplatin+paclitaxel BCP 400 atezolizumab+BCP ABCP 400 | 1202 | III | Open-label | NCT02366143 |
| 124 | Herbst, R. S., et al. (2020) | IV | first-line | atezolizumab 285 chemotherapy 287 | 572 | III | Open-label | NCT02409342 |
| 125 | Jotte, R., et al. (2020) | IV | first-line | atezolizumab+carboplatin+paclitaxel A+CP 338 atezolizumab+carboplatin+nab-paclitaxel A+CnP 343 carboplatin+nab-paclitaxel CnP 340 | 1021 | III | Open-label | NCT02367794 |
| 126 | Hida, T., et al. (2018) | advanced | second-line | Japanese atezolizumab arm 36  docetaxel arm 28 | 64 | III | Open-label | NCT02008227 |
| 127 | Fehrenbacher, L., et al. (2016) | advanced or metastatic | second-line and third-line | atezolizumab 144  docetaxel 143 | 287 | II | Open-label | NCT01903993 |
| 128 | Johnson, B. E., et al. (2013) | IIIB with malignant pleural effusion, IV, or recurrent | first-line | bevacizumab+placebo 373  bevacizumab+erlotinib 370 | 743 | IIIb | Double-blind | NCT00257608 |
| 129* | Barlesi, F., et al. (2018) | IIIB or IV or recurrent | second-line or later | avelumab 396  docetaxel 396 | 792 | III | Open-label | NCT02395172 |
| 130 | Digumarti, R., et al. (2014) | IIIB/IV | first-line | bavituximab+paclitaxel and carboplatin 49 | 49 | II | Open-label | NCT00687817 |
| 131 | Takeda, M., et al. (2016) | IIIB or IV or recurrent | second-line or later | West Japan Oncology Group 5910L docetaxel+Bevacizumab 50  docetaxel 50 | 100 | II | Open-label | NCT01351415 |
| 132 | Zhou, C., et al. (2015) | advanced, metastatic, or recurrent | first-line | bevacizumab+carboplatin+paclitaxel 138 Pl+CP+placebo+carboplatin+paclitaxel 138 | 276 | III | Double-blind | NCT01364012 |
| 133 | Yang, Y., et al. (2019) | advanced, metastatic, or recurrent | first-line | IBI305 224 bevacizumab 226 | 450 | III | Double-blind | NCT02954172 |
| 134 | Nishio, M., et al. (2021) | IV | first-line | atezolizumab+carboplatin or cisplatin+pemetrexed 292 carboplatin or cisplatin+pemetrexed 286 | 578 | III | Open-label | NCT02657434 |
| 135 | Borghaei, H., et al. (2021) | IIIB/IV | second-line or later | nivolumab 427 docetaxel 427 | 854 | III | Open-label | NCT01642004 NCT01673867 |
| 136* | Awad, M. M., et al. (2021) | IIIB/IV | first-line | pemetrexed+carboplatin+Pembrolizumab 60 pemetrexed+carboplatin 63 | 123 | II | Open-label | NCT02039674 |

*:14 trials with subgroup analysis.

**Reference**

1. Zhou, C., et al., *Pyrotinib in HER2-Mutant Advanced Lung Adenocarcinoma After Platinum-Based Chemotherapy: A Multicenter, Open-Label, Single-Arm, Phase II Study.* J Clin Oncol, 2020. **38**(24): p. 2753-2761.

2. Zhao, S., et al., *Efficacy and Tolerability of Erlotinib 100 mg/d vs. Gefitinib 250 mg/d in EGFR-Mutated Advanced Non-small Cell Lung Cancer (E100VG250): an Open-Label, Randomized, Phase 2 Study.* Frontiers in oncology, 2020. **10**.

3. Zhang, X., et al., *Efficacy and Safety of Apatinib Plus Vinorelbine in Patients With Wild-Type Advanced Non-Small Cell Lung Cancer After Second-Line Treatment Failure: A Nonrandomized Clinical Trial.* JAMA Netw Open, 2020. **3**(3): p. e201226.

4. Ramalingam, S.S., et al., *Overall Survival with Osimertinib in Untreated, EGFR-Mutated Advanced NSCLC.* N Engl J Med, 2020. **382**(1): p. 41-50.

5. Papadimitrakopoulou, V.A., et al., *Osimertinib versus platinum-pemetrexed for patients with EGFR T790M advanced NSCLC and progression on a prior EGFR-tyrosine kinase inhibitor: AURA3 overall survival analysis.* Annals of oncology : official journal of the european society for medical oncology, 2020. **31**(11): p. 1536‐1544.

6. Nishio, M., et al., *Final Overall Survival and Other Efficacy and Safety Results From ASCEND-3: Phase II Study of Ceritinib in ALKi-Naive Patients With ALK-Rearranged NSCLC.* J Thorac Oncol, 2020. **15**(4): p. 609-617.

7. Huber, R.M., et al., *Brigatinib in Crizotinib-Refractory ALK+ NSCLC: 2-Year Follow-up on Systemic and Intracranial Outcomes in the Phase 2 ALTA Trial.* Journal of Thoracic Oncology, 2020. **15**(3): p. 404-415.

8. Goldberg, S.B., et al., *Randomized Trial of Afatinib Plus Cetuximab Versus Afatinib Alone for First-Line Treatment of EGFR-Mutant Non-Small-Cell Lung Cancer: Final Results From SWOG S1403.* J Clin Oncol, 2020. **38**(34): p. 4076-4085.

9. Cho, J.H., et al., *Osimertinib for Patients With Non-Small-Cell Lung Cancer Harboring Uncommon EGFR Mutations: A Multicenter, Open-Label, Phase II Trial (KCSG-LU15-09).* J Clin Oncol, 2020. **38**(5): p. 488-495.

10. Reckamp, K.L., et al., *Phase II Trial of Cabozantinib Plus Erlotinib in Patients With Advanced Epidermal Growth Factor Receptor (EGFR)-Mutant Non-small Cell Lung Cancer With Progressive Disease on Epidermal Growth Factor Receptor Tyrosine Kinase Inhibitor Therapy: A California Cancer Consortium Phase II Trial (NCI 9303).* Front Oncol, 2019. **9**: p. 132.

11. Moro-Sibilot, D., et al., *Crizotinib in c-MET- or ROS1-positive NSCLC: results of the AcSé phase II trial.* Ann Oncol, 2019. **30**(12): p. 1985-1991.

12. Melosky, B., et al., *Selumetinib in patients receiving standard pemetrexed and platinum-based chemotherapy for advanced or metastatic KRAS wildtype or unknown non-squamous non-small cell lung cancer: A randomized, multicenter, phase II study. Canadian Cancer Trials Group (CCTG) IND.219.* Lung Cancer, 2019. **133**: p. 48-55.

13. Kelly, R.J., et al., *A phase III, randomized, open-label study of ASP8273 versus erlotinib or gefitinib in patients with advanced stage IIIB/IV non-small-cell lung cancer.* Annals of Oncology, 2019. **30**(7): p. 1127-1133.

14. Jiao, L., et al., *Chinese Herbal Medicine Combined With EGFR-TKI in EGFR Mutation-Positive Advanced Pulmonary Adenocarcinoma (CATLA): A Multicenter, Randomized, Double-Blind, Placebo-Controlled Trial.* Front Pharmacol, 2019. **10**: p. 732.

15. Wu, Y.L., et al., *Results of PROFILE 1029, a Phase III Comparison of First-Line Crizotinib versus Chemotherapy in East Asian Patients with ALK-Positive Advanced Non–Small Cell Lung Cancer.* Journal of Thoracic Oncology, 2018. **13**(10): p. 1539-1548.

16. Soria, J.C., et al., *Osimertinib in untreated EGFR-Mutated advanced non-small-cell lung cancer.* New England Journal of Medicine, 2018. **378**(2): p. 113-125.

17. Scagliotti, G.V., et al., *Tivantinib in Combination with Erlotinib versus Erlotinib Alone for EGFR-Mutant NSCLC: An Exploratory Analysis of the Phase 3 MARQUEE Study.* Journal of Thoracic Oncology, 2018. **13**(6): p. 849-854.

18. Mok, T.S., et al., *Improvement in Overall Survival in a Randomized Study That Compared Dacomitinib With Gefitinib in Patients With Advanced Non-Small-Cell Lung Cancer and EGFR-Activating Mutations.* J Clin Oncol, 2018. **36**(22): p. 2244-2250.

19. Lu, S., et al., *Randomized, Double-Blind, Placebo-Controlled, Multicenter Phase II Study of Fruquintinib After Two Prior Chemotherapy Regimens in Chinese Patients With Advanced Nonsquamous Non‒Small-Cell Lung Cancer.* J Clin Oncol, 2018. **36**(12): p. 1207-1217.

20. Han, B., et al., *Effect of Anlotinib as a Third-Line or Further Treatment on Overall Survival of Patients With Advanced Non-Small Cell Lung Cancer: The ALTER 0303 Phase 3 Randomized Clinical Trial.* JAMA Oncol, 2018. **4**(11): p. 1569-1575.

21. Garon, E.B., et al., *Randomized phase II study of fulvestrant and erlotinib compared with erlotinib alone in patients with advanced or metastatic non-small cell lung cancer.* Lung Cancer, 2018. **123**: p. 91-98.

22. Soria, J.C., et al., *First-line ceritinib versus platinum-based chemotherapy in advanced ALK-rearranged non-small-cell lung cancer (ASCEND-4): a randomised, open-label, phase 3 study.* The Lancet, 2017. **389**(10072): p. 917-929.

23. Soria, J.C., et al., *SELECT-2: A phase II, double-blind, randomized, placebo-controlled study to assess the efficacy of selumetinib plus docetaxel as a second-line treatment of patients with advanced or metastatic non-small-cell lung cancer.* Annals of Oncology, 2017. **28**(12): p. 3028-3036.

24. Lee, Y., et al., *Randomized phase II study of afatinib plus simvastatin versus afatinib alone in previously treated patients with advanced nonadenocarcinomatous non-small cell lung cancer.* Cancer Research and Treatment, 2017. **49**(4): p. 1001-1011.

25. Jänne, P.A., et al., *Selumetinib Plus Docetaxel Compared With Docetaxel Alone and Progression-Free Survival in Patients With KRAS-Mutant Advanced Non-Small Cell Lung Cancer: the SELECT-1 Randomized Clinical Trial.* JAMA, 2017. **317**(18): p. 1844‐1853.

26. Ciuleanu, T.E., et al., *Randomised Phase 2 study of maintenance linsitinib (OSI-906) in combination with erlotinib compared with placebo plus erlotinib after platinum-based chemotherapy in patients with advanced non-small cell lung cancer.* British Journal of Cancer, 2017. **117**(6): p. 757-766.

27. Yang, J.C., et al., *First-Line Pemetrexed plus Cisplatin followed by Gefitinib Maintenance Therapy versus Gefitinib Monotherapy in East Asian Never-Smoker Patients with Locally Advanced or Metastatic Nonsquamous Non-Small Cell Lung Cancer: Final Overall Survival Results from a Randomized Phase 3 Study.* J Thorac Oncol, 2016. **11**(3): p. 370-9.

28. Smit, E.F., et al., *A randomized, double-blind, phase III study comparing two doses of erlotinib for second-line treatment of current smokers with advanced non-small-cell lung cancer (CurrentS).* Lung Cancer, 2016. **99**: p. 94-101.

29. Schuler, M., et al., *First-Line Afatinib versus Chemotherapy in Patients with Non-Small Cell Lung Cancer and Common Epidermal Growth Factor Receptor Gene Mutations and Brain Metastases.* J Thorac Oncol, 2016. **11**(3): p. 380-90.

30. Park, K., et al., *First-Line Erlotinib Therapy Until and Beyond Response Evaluation Criteria in Solid Tumors Progression in Asian Patients With Epidermal Growth Factor Receptor Mutation-Positive Non-Small-Cell Lung Cancer: The ASPIRATION Study.* JAMA Oncol, 2016. **2**(3): p. 305-12.

31. Kim, Y.S., et al., *Randomized phase II study of pemetrexed versus gefitinib in previously treated patients with advanced non-small cell lung cancer.* Cancer Research and Treatment, 2016. **48**(1): p. 80-87.

32. Cheng, Y., et al., *Randomized phase II trial of gefitinib with and without pemetrexed as first-line therapy in patients with advanced nonsquamous non-small-cell lung cancer with activating epidermal growth factor receptor mutations.* Journal of Clinical Oncology, 2016. **34**(27): p. 3258-3266.

33. Zhong, W., et al., *Phase II study of biomarker-guided neoadjuvant treatment strategy for IIIA-N2 non-small cell lung cancer based on epidermal growth factor receptor mutation status.* J Hematol Oncol, 2015. **8**: p. 54.

34. Yoshioka, H., et al., *A randomized, double-blind, placebo-controlled, phase III trial of erlotinib with or without a c-Met inhibitor tivantinib (ARQ 197) in Asian patients with previously treated stage IIIB/IV nonsquamous nonsmall-cell lung cancer harboring wild-type epidermal growth factor receptor (ATTENTION study).* Annals of Oncology, 2015. **26**(10): p. 2066-2072.

35. Wu, Y.L., et al., *First-line erlotinib versus gemcitabine/cisplatin in patients with advanced EGFR mutation-positive non-small-cell lung cancer: analyses from the phase III, randomized, open-label, ENSURE study.* Ann Oncol, 2015. **26**(9): p. 1883-1889.

36. Soria, J.C., et al., *Gefitinib plus chemotherapy versus placebo plus chemotherapy in EGFR-mutation-positive non-small-cell lung cancer after progression on first-line gefitinib (IMPRESS): A phase 3 randomised trial.* The Lancet Oncology, 2015. **16**(8): p. 990-998.

37. Scagliotti, G., et al., *Phase III multinational, randomized, double-blind, placebo-controlled study of tivantinib (ARQ 197) plus erlotinib versus erlotinib alone in previously treated patients with locally advanced or metastatic nonsquamous non-small-cell lung cancer.* Journal of Clinical Oncology, 2015. **33**(24): p. 2667-2674.

38. Choi, Y.J., et al., *Randomized phase II study of paclitaxel/carboplatin intercalated with gefitinib compared to paclitaxel/carboplatin alone for chemotherapy-naïve non-small cell lung cancer in a clinically selected population excluding patients with non-smoking adenocarcinoma or mutated EGFR.* BMC Cancer, 2015. **15**(1).

39. Bondarenko, I.M., et al., *Phase II study of axitinib with doublet chemotherapy in patients with advanced squamous non-small-cell lung cancer.* BMC Cancer, 2015. **15**: p. 339.

40. Blumenschein, G.R., et al., *A randomized phase II study of the MEK1/MEK2 inhibitor trametinib (GSK1120212) compared with docetaxel in KRAS-mutant advanced non-small-cell lung cancer (NSCLC).* Annals of Oncology, 2015. **26**(5): p. 894-901.

41. Zhou, Q., et al., *Pemetrexed versus gefitinib as a second-line treatment in advanced nonsquamous nonsmall-cell lung cancer patients harboring wild-type EGFR (CTONG0806): A multicenter randomized trial.* Annals of Oncology, 2014. **25**(12): p. 2385-2391.

42. Solomon, B.J., et al., *First-line crizotinib versus chemotherapy in ALK-positive lung cancer.* New England Journal of Medicine, 2014. **371**(23): p. 2167-2177.

43. Karavasilis, V., et al., *Docetaxel and intermittent erlotinib in patients with metastatic non-small cell lung cancer; a phase II study from the hellenic cooperative oncology group.* Anticancer Research, 2014. **34**(10): p. 5649-5655.

44. Gitlitz, B.J., et al., *A randomized, placebo-controlled, multicenter, biomarker-selected, phase 2 study of apricoxib in combination with erlotinib in patients with advanced non-small-cell lung cancer.* J Thorac Oncol, 2014. **9**(4): p. 577-82.

45. Dittrich, C., et al., *A randomised phase II study of pemetrexed versus pemetrexed + erlotinib as second-line treatment for locally advanced or metastatic non-squamous non-small cell lung cancer.* European Journal of Cancer, 2014. **50**(9): p. 1571-1580.

46. Belani, C.P., et al., *Randomized phase II study of pemetrexed/cisplatin with or without axitinib for non-squamous non-small-cell lung cancer.* BMC Cancer, 2014. **14**(1).

47. Auliac, J.B., et al., *Randomized open-label non-comparative multicenter phase II trial of sequential erlotinib and docetaxel versus docetaxel alone in patients with non-small-cell lung cancer after failure of first-line chemotherapy: GFPC 10.02 study.* Lung Cancer, 2014. **85**(3): p. 415-419.

48. Katakami, N., et al., *LUX-Lung 4: a phase II trial of afatinib in patients with advanced non-small-cell lung cancer who progressed during prior treatment with erlotinib, gefitinib, or both.* J Clin Oncol, 2013. **31**(27): p. 3335-41.

49. Karampeazis, A., et al., *Pemetrexed versus erlotinib in pretreated patients with advanced non-small cell lung cancer: A Hellenic Oncology Research Group (HORG) randomized phase 3 study.* Cancer, 2013. **119**(15): p. 2754-2764.

50. Jänne, P.A., et al., *Selumetinib plus docetaxel for KRAS-mutant advanced non-small-cell lung cancer: A randomised, multicentre, placebo-controlled, phase 2 study.* The Lancet Oncology, 2013. **14**(1): p. 38-47.

51. Groen, H.J.M., et al., *A randomized, double-blind, phase ii study of erlotinib with or without sunitinib for the second-line treatment of metastatic non-small-cell lung cancer (NSCLC).* Annals of Oncology, 2013. **24**(9): p. 2382-2389.

52. Aerts, J.G., et al., *A randomized phase II study comparing erlotinib versus erlotinib with alternating chemotherapy in relapsed non-small-cell lung cancer patients: The NVALT-10 study.* Annals of Oncology, 2013. **24**(11): p. 2860-2865.

53. Zhang, L., et al., *Gefitinib versus placebo as maintenance therapy in patients with locally advanced or metastatic non-small-cell lung cancer (INFORM; C-TONG 0804): a multicentre, double-blind randomised phase 3 trial.* Lancet Oncol, 2012. **13**(5): p. 466-75.

54. Scagliotti, G.V., et al., *Rationale and design of MARQUEE: a phase III, randomized, double-blind study of tivantinib plus erlotinib versus placebo plus erlotinib in previously treated patients with locally advanced or metastatic, nonsquamous, non-small-cell lung cancer.* Clin Lung Cancer, 2012. **13**(5): p. 391-5.

55. Chen, Y.M., et al., *Phase II randomized trial of erlotinib or vinorelbine in chemonaive, advanced, non-small cell lung cancer patients aged 70 years or older.* Journal of Thoracic Oncology, 2012. **7**(2): p. 412-418.

56. Bauman, J.E., et al., *A Phase II study of pulse dose imatinib mesylate and weekly paclitaxel in patients aged 70 and over with advanced non-small cell lung cancer.* BMC Cancer, 2012. **12**: p. 449.

57. Ramalingam, S.S., et al., *Randomized phase II study of erlotinib in combination with placebo or R1507, a monoclonal antibody to insulin-like growth factor-1 receptor, for advanced-stage non-small-cell lung cancer.* Journal of Clinical Oncology, 2011. **29**(34): p. 4574-4580.

58. Natale, R.B., et al., *Phase III trial of vandetanib compared with erlotinib in patients with previously treated advanced non - small-cell lung cancer.* Journal of Clinical Oncology, 2011. **29**(8): p. 1059-1066.

59. Han, J.Y., et al., *A randomized phase II study of gefitinib plus simvastatin versus gefitinib alone in previously treated patients with advanced non-small cell lung cancer.* Clinical Cancer Research, 2011. **17**(6): p. 1553-1560.

60. Gaafar, R.M., et al., *A double-blind, randomised, placebo-controlled phase III intergroup study of gefitinib in patients with advanced NSCLC, non-progressing after first line platinum-based chemotherapy (EORTC 08021/ILCP 01/03).* European Journal of Cancer, 2011. **47**(15): p. 2331-2340.

61. Lee, D.H., et al., *Randomized phase III trial of gefitinib versus docetaxel in non-small cell lung cancer patients who have previously received platinum-based chemotherapy.* Clinical Cancer Research, 2010. **16**(4): p. 1307-1314.

62. Douillard, J.Y., et al., *Molecular predictors of outcome with gefitinib and docetaxel in previously treated non-small-cell lung cancer: Data from the randomized phase III INTEREST trial.* Journal of Clinical Oncology, 2010. **28**(5): p. 744-752.

63. Cappuzzo, F., et al., *Erlotinib as maintenance treatment in advanced non-small-cell lung cancer: a multicentre, randomised, placebo-controlled phase 3 study.* Lancet Oncol, 2010. **11**(6): p. 521-9.

64. Goss, G., et al., *Randomized phase II study of gefitinib compared with placebo in chemotherapy-naive patients with advanced non-small-cell lung cancer and poor performance status.* Journal of Clinical Oncology, 2009. **27**(13): p. 2253-2260.

65. Simon, G.R., et al., *Phase 2 trial of docetaxel and gefitinib in the first-line treatment of patients with advanced nonsmall-cell lung cancer (NSCLC) who are 70 years of age or older.* Cancer, 2008. **112**(9): p. 2021-9.

66. Maruyama, R., et al., *Phase III study, V-15-32, of gefitinib versus docetaxel in previously treated Japanese patients with non-small-cell lung cancer.* J Clin Oncol, 2008. **26**(26): p. 4244-52.

67. Crinò, L., et al., *Gefitinib versus vinorelbine in chemotherapy-naïve elderly patients with advanced non-small-cell lung cancer (invite): A randomized, phase II study.* Journal of Clinical Oncology, 2008. **26**(26): p. 4253-4260.

68. Park, K., et al., *Afatinib versus gefitinib as first-line treatment of patients with EGFR mutation-positive non-small-cell lung cancer (LUX-Lung 7): a phase 2B, open-label, randomised controlled trial.* Lancet Oncol, 2016. **17**(5): p. 577-89.

69. Wu, Y.L., et al., *Afatinib versus gemcitabine/cisplatin for first-line treatment of Chinese patients with advanced non-small-cell lung cancer harboring EGFR mutations: subgroup analysis of the LUX-Lung 6 trial.* Onco Targets Ther, 2018. **11**: p. 8575-8587.

70. Papadimitrakopoulou, V., et al., *The BATTLE-2 Study: A Biomarker-Integrated Targeted Therapy Study in Previously Treated Patients With Advanced Non-Small-Cell Lung Cancer.* J Clin Oncol, 2016. **34**(30): p. 3638-3647.

71. Wu, Y.L., et al., *Randomized clinical trial of pembrolizumab vs chemotherapy for previously untreated Chinese patients with PD-L1-positive locally advanced or metastatic non-small-cell lung cancer: KEYNOTE-042 China Study.* Int J Cancer, 2021. **148**(9): p. 2313-2320.

72. Gadgeel, S., et al., *Updated Analysis From KEYNOTE-189: Pembrolizumab or Placebo Plus Pemetrexed and Platinum for Previously Untreated Metastatic Nonsquamous Non-Small-Cell Lung Cancer.* J Clin Oncol, 2020. **38**(14): p. 1505-1517.

73. Cortot, A.B., et al., *Weekly paclitaxel plus bevacizumab versus docetaxel as second- or third-line treatment in advanced non-squamous non-small-cell lung cancer: Results of the IFCT-1103 ULTIMATE study.* Eur J Cancer, 2020. **131**: p. 27-36.

74. Chen, Y.M., et al., *Nivolumab safety and efficacy in advanced, platinum-resistant, non-small cell lung cancer, radical radiotherapy-ineligible patients: A phase II study in Taiwan.* J Formos Med Assoc, 2020. **119**(12): p. 1817-1826.

75. Arrieta, O., et al., *Efficacy and Safety of Pembrolizumab Plus Docetaxel vs Docetaxel Alone in Patients With Previously Treated Advanced Non-Small Cell Lung Cancer: The PROLUNG Phase 2 Randomized Clinical Trial.* JAMA Oncol, 2020. **6**(6): p. 856-864.

76. Wu, Y.L., et al., *Nivolumab Versus Docetaxel in a Predominantly Chinese Patient Population With Previously Treated Advanced NSCLC: CheckMate 078 Randomized Phase III Clinical Trial.* J Thorac Oncol, 2019. **14**(5): p. 867-875.

77. Villaruz, L.C., et al., *A phase II study of nab-paclitaxel and carboplatin chemotherapy plus necitumumab in the first-line treatment of patients with stage IV squamous non-small cell lung cancer.* Lung Cancer, 2019. **136**: p. 52-56.

78. Theelen, W.S.M.E., et al., *Effect of Pembrolizumab after Stereotactic Body Radiotherapy vs Pembrolizumab Alone on Tumor Response in Patients with Advanced Non-Small Cell Lung Cancer: Results of the PEMBRO-RT Phase 2 Randomized Clinical Trial.* JAMA Oncology, 2019. **5**(9): p. 1276-1282.

79. Stinchcombe, T.E., et al., *Effect of Erlotinib Plus Bevacizumab vs Erlotinib Alone on Progression-Free Survival in Patients With Advanced EGFR-Mutant Non-Small Cell Lung Cancer: A Phase 2 Randomized Clinical Trial.* JAMA Oncol, 2019. **5**(10): p. 1448-1455.

80. Sequist, L.V., et al., *Randomized Phase II Trial of Seribantumab in Combination with Erlotinib in Patients with EGFR Wild-Type Non-Small Cell Lung Cancer.* Oncologist, 2019. **24**(8): p. 1095-1102.

81. Reck, M., et al., *Updated Analysis of KEYNOTE-024: Pembrolizumab Versus Platinum-Based Chemotherapy for Advanced Non-Small-Cell Lung Cancer With PD-L1 Tumor Proportion Score of 50% or Greater.* J Clin Oncol, 2019. **37**(7): p. 537-546.

82. Patil, P.D., et al., *An Open-Label Phase II Trial of Bevacizumab plus Docetaxel and Gemcitabine in Advanced, Previously Untreated Nonsquamous Non-Small Cell Lung Cancer.* Oncologist, 2019. **24**(4): p. 457-e126.

83. Levy, B.P., et al., *Randomised phase 2 study of pembrolizumab plus CC-486 versus pembrolizumab plus placebo in patients with previously treated advanced non-small cell lung cancer.* Eur J Cancer, 2019. **108**: p. 120-128.

84. Herbst, R.S., et al., *Use of archival versus newly collected tumor samples for assessing PD-L1 expression and overall survival: an updated analysis of KEYNOTE-010 trial.* Ann Oncol, 2019. **30**(2): p. 281-289.

85. von Pawel, J., et al., *Randomized Phase II Trial of Parsatuzumab (Anti-EGFL7) or Placebo in Combination with Carboplatin, Paclitaxel, and Bevacizumab for First-Line Nonsquamous Non-Small Cell Lung Cancer.* Oncologist, 2018. **23**(6): p. 654-e58.

86. Spigel, D.R., et al., *Randomized phase 2 trial of pemetrexed, pemetrexed/bevacizumab, and pemetrexed/carboplatin/bevacizumab in patients with stage IIIB/IV non-small cell lung cancer and an Eastern Cooperative Oncology Group performance status of 2.* Cancer, 2018. **124**(9): p. 1982-1991.

87. Paz-Ares, L., et al., *Pembrolizumab plus Chemotherapy for Squamous Non-Small-Cell Lung Cancer.* N Engl J Med, 2018. **379**(21): p. 2040-2051.

88. Nie, K., et al., *Osimertinib compared docetaxel-bevacizumab as third-line treatment in EGFR T790M mutated non-small-cell lung cancer.* Lung Cancer, 2018. **121**: p. 5-11.

89. Nakao, A., et al., *Nab-paclitaxel maintenance therapy following carboplatin + nab-paclitaxel combination therapy in chemotherapy naïve patients with advanced non-small cell lung cancer: multicenter, open-label, single-arm phase II trial.* Invest New Drugs, 2018. **36**(5): p. 903-910.

90. Marrone, K.A., et al., *A Randomized Phase II Study of Metformin plus Paclitaxel/Carboplatin/Bevacizumab in Patients with Chemotherapy-Naïve Advanced or Metastatic Nonsquamous Non-Small Cell Lung Cancer.* Oncologist, 2018. **23**(7): p. 859-865.

91. Herbst, R.S., et al., *Cetuximab plus carboplatin and paclitaxel with or without bevacizumab versus carboplatin and paclitaxel with or without bevacizumab in advanced NSCLC (SWOG S0819): a randomised, phase 3 study.* Lancet Oncol, 2018. **19**(1): p. 101-114.

92. Gridelli, C., et al., *Safety and Efficacy of Bevacizumab Plus Standard-of-Care Treatment Beyond Disease Progression in Patients With Advanced Non-Small Cell Lung Cancer: The AvaALL Randomized Clinical Trial.* JAMA Oncol, 2018. **4**(12): p. e183486.

93. Gandhi, L., et al., *Pembrolizumab plus Chemotherapy in Metastatic Non-Small-Cell Lung Cancer.* N Engl J Med, 2018. **378**(22): p. 2078-2092.

94. Engel-Riedel, W., et al., *A randomized, controlled trial evaluating the efficacy and safety of BTH1677 in combination with bevacizumab, carboplatin, and paclitaxel in first-line treatment of advanced non-small cell lung cancer.* J Immunother Cancer, 2018. **6**(1): p. 16.

95. Ciuleanu, T., et al., *Efficacy and Safety of Necitumumab Continuation Therapy in the Phase III SQUIRE Study of Patients With Stage IV Squamous Non-Small-Cell Lung Cancer.* Clin Lung Cancer, 2018. **19**(2): p. 130-138.e2.

96. Antonia, S.J., et al., *Durvalumab after Chemoradiotherapy in Stage III Non-Small-Cell Lung Cancer.* N Engl J Med, 2017. **377**(20): p. 1919-1929.

97. Spigel, D.R., et al., *An Open-Label, Randomized, Controlled Phase II Study of Paclitaxel-Carboplatin Chemotherapy With Necitumumab Versus Paclitaxel-Carboplatin Alone in First-Line Treatment of Patients With Stage IV Squamous Non–Small-Cell Lung Cancer.* Clinical Lung Cancer, 2017. **18**(5): p. 480-488.

98. Novello, S., et al., *An Open-Label, Multicenter, Randomized, Phase II Study of Cisplatin and Pemetrexed With or Without Cixutumumab (IMC-A12) as a First-Line Therapy in Patients With Advanced Nonsquamous Non-Small Cell Lung Cancer.* J Thorac Oncol, 2017. **12**(2): p. 383-389.

99. Kim, H.R., et al., *A randomized, phase II study of gefitinib alone versus nimotuzumab plus gefitinib after platinum-based chemotherapy in advanced non-small cell lung cancer (KCSG LU12-01).* Oncotarget, 2017. **8**(9): p. 15943-15951.

100. Gerber, D.E., et al., *Phase II study of olaratumab with paclitaxel/carboplatin (P/C) or P/C alone in previously untreated advanced NSCLC.* Lung Cancer, 2017. **111**: p. 108-115.

101. Carbone, D.P., et al., *First-line nivolumab in stage IV or recurrent non-small-cell lung cancer.* New England Journal of Medicine, 2017. **376**(25): p. 2415-2426.

102. Yoh, K., et al., *A randomized, double-blind, phase II study of ramucirumab plus docetaxel vs placebo plus docetaxel in Japanese patients with stage IV non-small cell lung cancer after disease progression on platinum-based therapy.* Lung Cancer, 2016. **99**: p. 186-193.

103. Reck, M., et al., *Pembrolizumab versus Chemotherapy for PD-L1-Positive Non-Small-Cell Lung Cancer.* New England Journal of Medicine, 2016. **375**(19): p. 1823-1833.

104. Zinner, R.G., et al., *PRONOUNCE: Randomized, open-label, phase III study of first-line pemetrexed + carboplatin followed by maintenance pemetrexed versus paclitaxel + carboplatin + bevacizumab followed by maintenance bevacizumab in patients ith advanced nonsquamous non-small-cell lung cancer.* Journal of Thoracic Oncology, 2015. **10**(1): p. 134-142.

105. Thomas, M., et al., *Erlotinib and bevacizumab versus cisplatin, gemcitabine and bevacizumab in unselected nonsquamous nonsmall cell lung cancer.* European Respiratory Journal, 2015. **46**(1): p. 219-229.

106. Scagliotti, G.V., et al., *Randomized, phase III trial of figitumumab in combination with erlotinib versus erlotinib alone in patients with nonadenocarcinoma nonsmall-cell lung cancer.* Annals of Oncology, 2015. **26**(3): p. 497-504.

107. Kotsakis, A., et al., *Sequential administration of vinorelbine plus cisplatin and bevacizumab followed by docetaxel plus gemcitabine and bevacizumab compared to docetaxel plus cisplatin and bevacizumab regimen as first-line therapy for advanced or metastatic non-squamous non-small cell lung cancer: A multicenter randomized phase II trial of the Hellenic Oncology Research Group (HORG).* Lung Cancer, 2015. **88**(1): p. 57-62.

108. Dingemans, A.M., et al., *A randomized phase II study comparing paclitaxel-carboplatin-bevacizumab with or without nitroglycerin patches in patients with stage IV nonsquamous nonsmall-cell lung cancer: NVALT12 (NCT01171170)†.* Ann Oncol, 2015. **26**(11): p. 2286-93.

109. Brahmer, J., et al., *Nivolumab versus docetaxel in advanced squamous-cell non-small-cell lung cancer.* New England Journal of Medicine, 2015. **373**(2): p. 123-135.

110. Borghaei, H., et al., *Nivolumab versus docetaxel in advanced nonsquamous non-small-cell lung cancer.* New England Journal of Medicine, 2015. **373**(17): p. 1627-1639.

111. Paz-Ares, L., et al., *Necitumumab plus pemetrexed and cisplatin as first-line therapy in patients with stage IV non-squamous non-small-cell lung cancer (INSPIRE): an open-label, randomised, controlled phase 3 study.* The lancet. Oncology, 2015. **16**(3): p. 328‐337.

112. Twelves, C., et al., *Randomised phase II study of axitinib or bevacizumab combined with paclitaxel/carboplatin as first-line therapy for patients with advanced non-small-cell lung cancer.* Annals of Oncology, 2014. **25**(1): p. 132-138.

113. Reck, M., et al., *Docetaxel plus nintedanib versus docetaxel plus placebo in patients with previously treated non-small-cell lung cancer (LUME-Lung 1): A phase 3, double-blind, randomised controlled trial.* The Lancet Oncology, 2014. **15**(2): p. 143-155.

114. Langer, C.J., et al., *Randomized, phase III trial of first-line figitumumab in combination with paclitaxel and carboplatin versus paclitaxel and carboplatin alone in patients with advanced non-small-cell lung cancer.* Journal of Clinical Oncology, 2014. **32**(19): p. 2059-2066.

115. Garon, E.B., et al., *Ramucirumab plus docetaxel versus placebo plus docetaxel for second-line treatment of stage IV non-small-cell lung cancer after disease progression on platinum-based therapy (REVEL): A multicentre, double-blind, randomised phase 3 trial.* The Lancet, 2014. **384**(9944): p. 665-673.

116. Reck, M., et al., *A randomized, double-blind, placebo-controlled phase 2 study of tigatuzumab (CS-1008) in combination with carboplatin/paclitaxel in patients with chemotherapy-naïve metastatic/unresectable non-small cell lung cancer.* Lung Cancer, 2013. **82**(3): p. 441-448.

117. Patel, J.D., et al., *PointBreak: a randomized phase III study of pemetrexed plus carboplatin and bevacizumab followed by maintenance pemetrexed and bevacizumab versus paclitaxel plus carboplatin and bevacizumab followed by maintenance bevacizumab in patients with stage IIIB or IV nonsquamous non-small-cell lung cancer.* J Clin Oncol, 2013. **31**(34): p. 4349-57.

118. Ciuleanu, T., et al., *A phase II study of erlotinib in combination with bevacizumab versus chemotherapy plus bevacizumab in the first-line treatment of advanced non-squamous non-small cell lung cancer.* Lung Cancer, 2013. **82**(2): p. 276-81.

119. Garon, E.B., et al., *A randomized, double-blind, phase III study of docetaxel and ramucirumab versus docetaxel and placebo in the treatment of stage IV non-small-cell lung cancer after disease progression after 1 previous platinum-based therapy (REVEL): Treatment rationale and study design.* Clinical Lung Cancer, 2012. **13**(6): p. 505-509.

120. Herbst, R.S., et al., *Efficacy of bevacizumab plus erlotinib versus erlotinib alone in advanced non-small-cell lung cancer after failure of standard first-line chemotherapy (BeTa): A double-blind, placebo-controlled, phase 3 trial.* The Lancet, 2011. **377**(9780): p. 1846-1854.

121. Blumenschein, G.R., et al., *A phase II, multicenter, open-label randomized study of motesanib or bevacizumab in combination with paclitaxel and carboplatin for advanced nonsquamous non-small-cell lung cancer.* Annals of Oncology, 2011. **22**(9): p. 2057-2067.

122. Planchard, D., et al., *ARCTIC: durvalumab with or without tremelimumab as third-line or later treatment of metastatic non-small-cell lung cancer.* Ann Oncol, 2020. **31**(5): p. 609-618.

123. Socinski, M.A., et al., *Atezolizumab for First-Line Treatment of Metastatic Nonsquamous NSCLC.* N Engl J Med, 2018. **378**(24): p. 2288-2301.

124. Herbst, R.S., et al., *Atezolizumab for First-Line Treatment of PD-L1-Selected Patients with NSCLC.* N Engl J Med, 2020. **383**(14): p. 1328-1339.

125. Jotte, R., et al., *Atezolizumab in Combination With Carboplatin and Nab-Paclitaxel in Advanced Squamous NSCLC (IMpower131): Results From a Randomized Phase III Trial.* J Thorac Oncol, 2020. **15**(8): p. 1351-1360.

126. Hida, T., et al., *Atezolizumab in Japanese Patients With Previously Treated Advanced Non-Small-Cell Lung Cancer: A Subgroup Analysis of the Phase 3 OAK Study.* Clin Lung Cancer, 2018. **19**(4): p. e405-e415.

127. Fehrenbacher, L., et al., *Atezolizumab versus docetaxel for patients with previously treated non-small-cell lung cancer (POPLAR): a multicentre, open-label, phase 2 randomised controlled trial.* Lancet, 2016. **387**(10030): p. 1837-46.

128. Johnson, B.E., et al., *ATLAS: randomized, double-blind, placebo-controlled, phase IIIB trial comparing bevacizumab therapy with or without erlotinib, after completion of chemotherapy, with bevacizumab for first-line treatment of advanced non-small-cell lung cancer.* J Clin Oncol, 2013. **31**(31): p. 3926-34.

129. Barlesi, F., et al., *Avelumab versus docetaxel in patients with platinum-treated advanced non-small-cell lung cancer (JAVELIN Lung 200): an open-label, randomised, phase 3 study.* Lancet Oncol, 2018. **19**(11): p. 1468-1479.

130. Digumarti, R., et al., *Bavituximab plus paclitaxel and carboplatin for the treatment of advanced non-small-cell lung cancer.* Lung Cancer, 2014. **86**(2): p. 231-6.

131. Takeda, M., et al., *Bevacizumab beyond disease progression after first-line treatment with bevacizumab plus chemotherapy in advanced nonsquamous non-small cell lung cancer (West Japan Oncology Group 5910L): An open-label, randomized, phase 2 trial.* Cancer, 2016. **122**(7): p. 1050-9.

132. Zhou, C., et al., *BEYOND: A Randomized, Double-Blind, Placebo-Controlled, Multicenter, Phase III Study of First-Line Carboplatin/Paclitaxel Plus Bevacizumab or Placebo in Chinese Patients With Advanced or Recurrent Nonsquamous Non-Small-Cell Lung Cancer.* J Clin Oncol, 2015. **33**(19): p. 2197-204.

133. Yang, Y., et al., *Biosimilar candidate IBI305 plus paclitaxel/carboplatin for the treatment of non-squamous non-small cell lung cancer.* Transl Lung Cancer Res, 2019. **8**(6): p. 989-999.

134. Nishio, M., et al., *Atezolizumab Plus Chemotherapy for First-Line Treatment of Nonsquamous NSCLC: Results From the Randomized Phase 3 IMpower132 Trial.* J Thorac Oncol, 2021. **16**(4): p. 653-664.

135. Borghaei, H., et al., *Five-Year Outcomes From the Randomized, Phase III Trials CheckMate 017 and 057: Nivolumab Versus Docetaxel in Previously Treated Non-Small-Cell Lung Cancer.* J Clin Oncol, 2021. **39**(7): p. 723-733.

136. Awad, M.M., et al., *Long-Term Overall Survival From KEYNOTE-021 Cohort G: Pemetrexed and Carboplatin With or Without Pembrolizumab as First-Line Therapy for Advanced Nonsquamous NSCLC.* J Thorac Oncol, 2021. **16**(1): p. 162-168.
